# LNMAC Promotes Cervical Squamous Cell Carcinoma Lymphatic Metastasis via Epigenetic Regulation of FGF2‐Induced Lymphangiogenesis

**DOI:** 10.1002/advs.202404645

**Published:** 2024-08-09

**Authors:** Chunyu Zhang, Li Yuan, Weijia Wen, Caixia Shao, Yuandong Liao, Yan Jia, Xueyuan Zhao, Yan Liao, Dingze Xu, Linna Chen, Guofen Yang, Hongye Jiang, Wei Wang, Shuzhong Yao

**Affiliations:** ^1^ Department of Obstetrics and Gynecology the First Affiliated Hospital Sun Yat‐sen University Guangzhou Guangdong 510080 P. R. China; ^2^ Guangdong Provincial Clinical Research Center for Obstetrical and Gynecological Diseases Guangzhou Guangdong 510080 P. R. China

**Keywords:** cervical squamous cell carcinoma, FGF2, LNMAC, lymphangiogenesis, lymph node metastasis

## Abstract

The lymph node is the most common site of distant metastasis of cervical squamous cell carcinoma (CSCC), which elicits dismal prognosis and limited efficiency for treatment. Elucidation of the mechanisms underlying CSCC lymphatic metastasis would provide potential therapeutic strategies for nodal metastatic of CSCC. Here, based on in vivo lymphatic metastasis screening model, a circular RNA is identified that is termed as lymph node metastasis associated circRNA (LNMAC), is markedly upregulated in lymphatic metastatic CSCC and correlated with lymph node metastasis. Overexpression of LNMAC dramatically augments the metastatic capability of CSCC cells to the lymph node via inducing lymphangiogenesis. Mechanistically, LNMAC epigenetically upregulates fibroblast growth factor 2 (FGF2) expression by directly associating with histone acacetylase 1 (HDAC1), preventing Importin α6/8‐mediated nuclear translocation of HDAC1 and eliciting histone H3K27ac‐induced FGF2 transcriptional activation. Treatment with 3F12E7, an anti‐FGF2 monoclonal antibody, effectively inhibits LNMAC‐induced CSCC lymphatic metastasis. Taken together, these findings indicate that LNMAC plays a crucial role in FGF2‐mediated lymphangiogenesis and lymphatic metastasis, highlighting that LNMAC might be a therapeutic target for lymph node metastasis in CSCC patients.

## Graphical abstract

Cervical squamous cell carcinoma (CSCC) cells mainly metastasize to the lymph nodes. This study reveals that LNMAC dramatically augments CSCC lymphangiogenesis and lymphatic metastasis by binding with histone deacetylase 1 (HDAC1), preventing Importin α6/8‐mediated HDAC1 nuclear translocation and eliciting histone H3K27ac‐induced fibroblast growth factor 2 (FGF2) transcriptional activation. Treatment with 3F12E7 effectively inhibits LNMAC‐induced CSCC lymphatic metastasis in preclinical models.

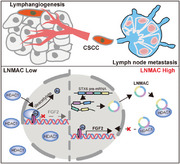

## 1 Introduction

Cervical cancer is one of the most common female cancers worldwide.^[^ ^1^ ^]^ Approximately 70% to 80% of cervical cancers are cervical squamous cell carcinomas (CSCC), 20% to 25% are adenocarcinomas, and the rest are adenosquamous and rare histological types.^[^ ^2^, ^3^, ^4^, ^5^ ^]^ Due to the unique pelvic anatomy and their innate pathological characteristics, CSCC cells mainly metastasize to the pelvic and para‐aortic lymph nodes (LN) via lymphatic vessels.^[^ ^6^ ^]^ Although patients with early stage CSCC undergoing surgical treatment can achieve a favorable prognosis, the 5‐year overall survival rate associated with lymph node metastasis (LNM) is unsatisfactory. The presence of lymph node metastasis itself decreases the 5‐year survival rate from 95% to 33.3%.^[^ ^7^, ^8^ ^]^ The updated 2018 International Federation of Gynecology and Obstetrics (FIGO) staging system of CSCC recommended that patients with LNM should be diagnosed with stage IIIC,^[^ ^9^ ^]^ suggesting that LNM status has a critical role in the prognosis of patients with CSCC. Given the mounting evidence suggesting that lymph nodes are the foothold for further tumor dissemination, elucidation of the mechanisms underlying LNM in CSCC is of paramount importance.

Lymph node metastasis is a complicated biological process involving intratumoral and peritumoral lymphangiogenesis in the primary tumor, invasiveness of tumor cells, entry of tumor cells into the lymphatic system, colonization, and expansion in metastatic LNs. It has been shown that lymphangiogenesis is a critical initiator of tumor lymphatic metastasis.^[^ ^10^, ^11^ ^]^ Accumulating evidence has demonstrated that upregulation of fibroblast growth factor 2 (FGF2), a lymphangiogenic growth factor, positively correlates with regional LN metastasis and poor survival in multiple human malignancies including liver cancer,^[^ ^12^ ^]^ melanoma^[^ ^13^ ^]^ and CSCC.^[^ ^14^ ^]^ Previous research has indicated that inhibition of FGF2‐mediated signaling pathways using siRNA, anti‐FGF2 antibodies, or “FGF ligand trap” neutralizing antibodies can halt metastatic spread of human malignancies. Importantly, FP‐1039 (GSK3052230), a FGF2 trap molecule, was tested in a clinical phase IB trial for patients with malignant pleural mesothelioma (ClinicalTrials.gov NCT01868022),^[^ ^15^ ^]^ highlighting that the road leading to development of FGF2 blocking molecules is still rugged but promising. Therefore, understanding the regulatory mechanism of FGF2 in CSCC may provide clinically valuable predictive tools for effective anti‐FGF2 treatments.

Circular RNAs (circRNAs) are generated from back‐splicing of pre‐mRNAs to form covalently closed transcripts.^[^ ^16^, ^17^ ^]^ CircRNA expression is conserved among species and highly cell‐type and tissue specific. Accumulating evidence indicates that circRNAs may be involved in metastasis of many cancers including bladder,^[^ ^18^ ^]^ lung^[^ ^19^ ^]^ and cervical.^[^ ^20^, ^21^ ^]^ We have previously reported that a circular RNA (circVPRBP) can inhibit cervical cancer LN metastasis through alteration of RACK1 O‐GlcNAcylation.^[^ ^20^ ^]^ Of note, circRNA‐mediated attenuation of FGF2 expression is expected for at least some of its regulation and biological function in cancer cells. Moreover, the precise mechanism and clinical significance of circRNAs in lymphangiogenesis and nodal metastasis of CSCC remain largely unknown, warranting further exploration.

In the present study, through in vivo lymphatic metastasis screening, we demonstrate that LNMAC (lymph node metastasis associated circRNA) overexpression, which has been correlated with CSCC nodal metastasis, suppresses the recruitment of HDAC1 to FGF2 promoters. LNMAC prevents Importin α6/8‐mediated nuclear translocation of HDAC1 through circRNA–protein interaction, eliciting histone H3K27ac‐induced transcriptional activation of FGF2, resulting in lymphangiogenesis and CSCC lymphatic metastasis. Therefore, our results indicate that LNMAC plays a crucial role in FGF2‐mediated lymphangiogenesis and lymphatic metastasis, highlighting that LNMAC might be a therapeutic target for LN metastasis in CSCC patients.

## 2 Results

### 2.1 LNMAC Is Upregulated in Lymph Node Metastatic CSCC Cells

**Figure 1:**
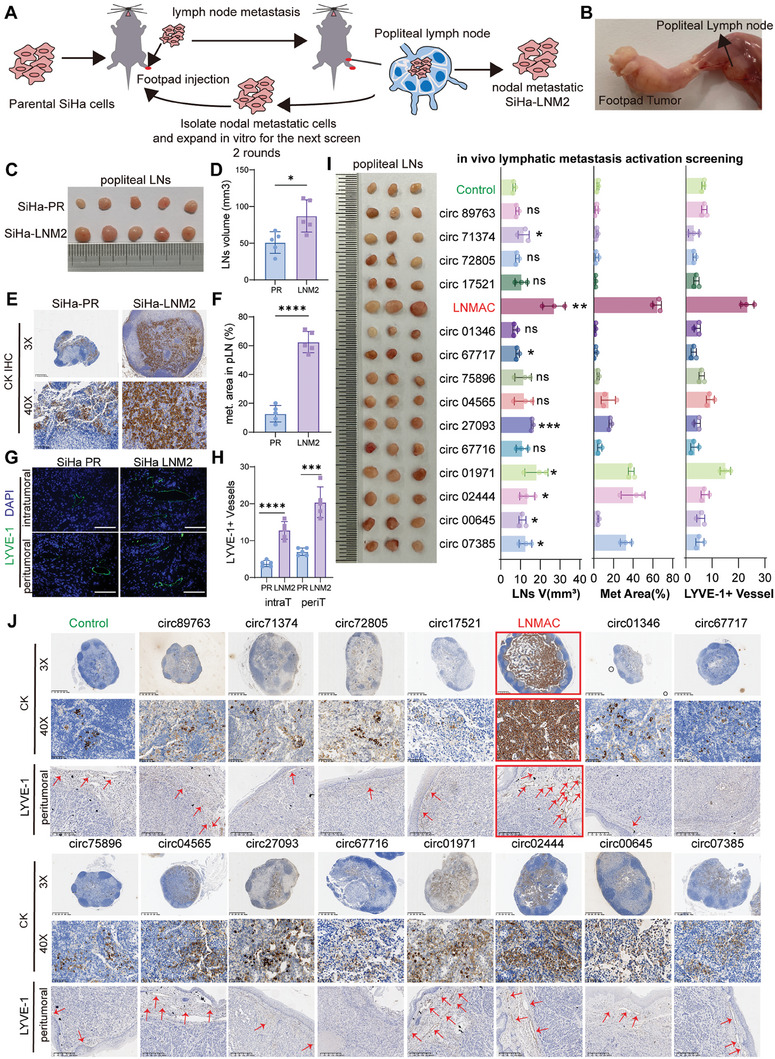
LNMAC is upregulated in lymph node metastatic CSCC cells. A) The schematic illustration of the screening of highly metastatic CSCC cells. B) The schematic illustration of in vivo nude mouse LN metastasis model of CSCC. C,D) Representative images and the volume of popliteal LNs in different groups (*n* = 5). E) Representative images of immunostaining of pan‐cytokeratin of popliteal LNs (*n* = 5). F) Metastatic area of popliteal LNs in different groups (*n* = 5). G,H) Representative images of immunofluorescence of LYVE‐1 and density of LYVE positive vessels in the indicated groups (*n* = 5). I) Representative images of popliteal LNs and the statistic results of LNs volume(mm^3^), metastatic area (%), and number of LYVE+ vessels in different groups (*n* = 3). J) Immunostaining of pan‐cytokeratin of popliteal LNs and the LYVE positive vessels in the peritumoral regions in different group. Red arrow represents LYVE positive vessels. ns no significance; *P < 0.05; ***P < 0.001; ****P < 0.0001.

To investigate the mechanism underlying CSCC lymph node metastasis, highly lymphatic metastatic CSCC cells were established using a lymph node metastatic mouse model after two rounds of footpad injection. As shown in the schematic in **Figure** **1**A, SiHa cells were chosen given their HPV‐positivity and derivation from primary CSCC (nonmetastatic organ origin) (Figure 1A). We used a nude mouse popliteal LNM model to simulate the pelvic linear drainage of the lymphatic system in CSCC (Figure 1B). The SiHa‐parental cells, which stably expressed a puromycin resistance element, were inoculated into the footpad of immunodeficient mice, and the formed metastatic cells were recovered from the popliteal lymph node. These cells were screened by the addition of puromycin (2 µg mL^−1^), expanded in culture, and reinjected into mice for the next round. After two rounds of in vivo selection, a lymph node‐metastatic subpopulation of SiHa cells was established and called SiHa‐LNM2. We assessed the LNM capacity of SiHa‐LNM2 cells compared to their parental counterpart (SiHa‐PR) in vivo. We found that the volume of popliteal lymph nodes was significantly larger in the SiHa‐LNM2 group than in the parental SiHa cells group (Figure 1C,D). Meanwhile, immunostaining of pan‐cytokeratin was used to analyze the metastatic area of the popliteal lymph nodes. SiHa‐LNM2 cells had a remarkably increased lymphatic metastatic area when compared to the corresponding parental cell line (Figure 1E,F). Moreover, SiHa‐LNM2 cells significantly enhanced LYVE‐1 positive vessel formation in both the intratumoral and peritumoral areas of the primary footpad tumor (Figure 1G,H). SiHa‐LNM2 cells distinctly promoted HLECs tube formation and migration in vivo when compared to SiHa‐PR cells (Figure S1A–D, Supporting Information), suggesting a prolymphangiogenesis and lymphatic metastatic effect of SiHa‐LNM2 cells. To identify critical factors that contribute to CSCC lymph node metastasis, circRNA microarray was performed using SiHa‐LNM2 and SiHa‐parental cells. After filtering differentially expressed circRNAs, we subjected the top 15 upregulated circRNAs (fold change (FC)>1.5 and *P* < 0.05) (Figures S2 and S3, Supporting Information) to in vivo lymphatic metastasis activation screening, which analyzed the volume of LNs, metastatic area, and the lymphatic vascular density (LVD). After lymphatic metastasis activation screening, LNMAC (lymph node metastasis associated circRNA, circbase ID: hsa_circ_0007905) was identified as the most promoting nodal metastatic factor among the aforementioned 15 circRNAs.

### 2.2 Characterization of LNMAC in CSCC Cells

**Figure 2:**
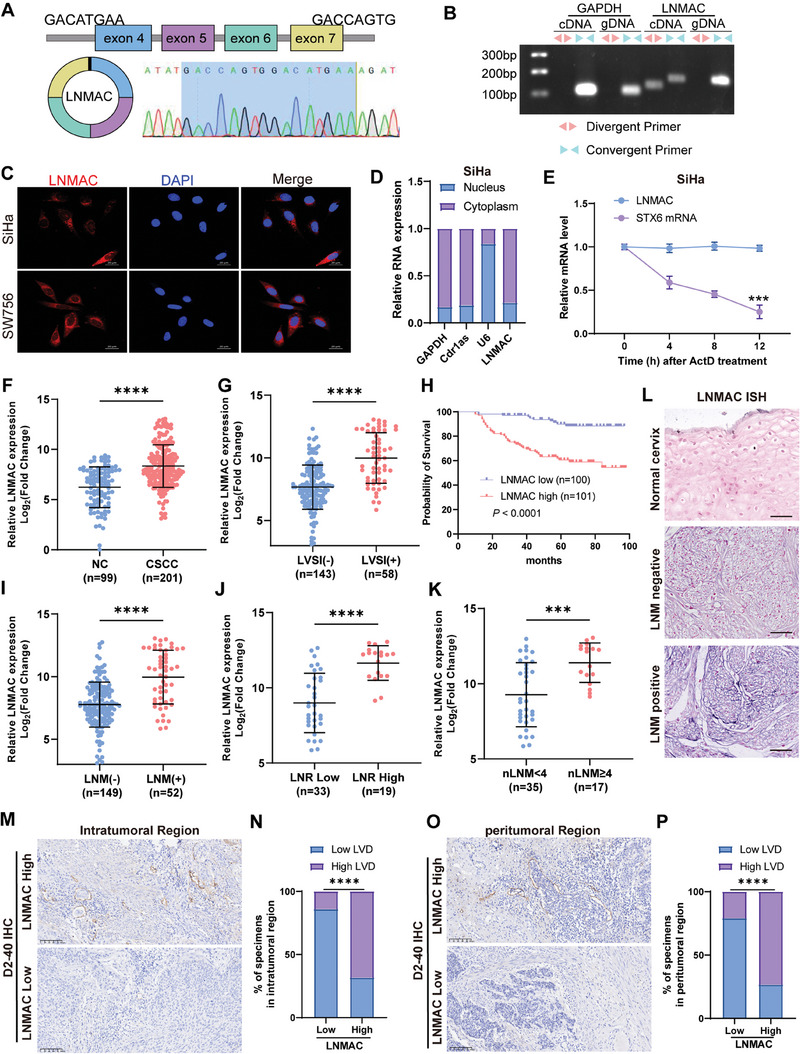
Characterization of LNMAC in CSCC cells. A) The genomic locus of LNMAC. The back splicing junction was identified by Sanger sequencing. B) PCR with agarose gel electrophoresis assay indicated the presence of LNMAC using convergent and divergent primers from cDNA or genomic DNA in CSCC cells. C) Representative FISH images showed LNMAC was mainly located in the cytoplasm in SiHa and SW756 cells. D) The location of LNMAC was confirmed using a subcellular fractionation assay. GAPDH and Cdr1as were used as controls which were mainly located in cytoplasm, while U6 was used as control which was mainly located in nucleus. E) Actinomycin D assay was used to evaluate the stability of LNMAC and STX6 mRNA in SiHa cells (*n* = 3). F) RT‐qPCR analysis of LNMAC expression in normal cervix tissues (*n* = 99) and CSCC samples (*n* = 201). G) RT‐qPCR analysis of LNMAC expression in LVSI negative (*n* = 143) and LVSI positive (n = 58) CSCC samples. H) Kaplan–Meier analysis showed the positive correlation between LNMAC expression levels and the overall survival in our cohort. I) RT‐qPCR analysis of LNMAC expression in CSCC samples with (*n* = 52) or without (*n* = 149) lymph node metastasis. J) RT‐qPCR analysis of LNMAC expression in LNR low (*n* = 33) and LNR high (*n* = 19) CSCC samples. K) RT‐qPCR analysis of LNMAC expression in CSCC sample with different number of metastatic LNs. L) Representative ISH images of LNMAC in different groups. M–P) Representative images of immunostaining of D2‐40 in the indicated groups. Each experiment was performed at least three times independently. ****P < 0.0001.

Our bioinformatic analysis indicated that LNMAC arises from exons 4 to 7 of the STX6 gene and is 391 nucleotides in length. Sanger sequencing showed the back‐splicing site of the LNMAC (**Figure** **2**A), which was experimentally confirmed by RT‐PCR using divergent primers (Figure 2B) and consistent with the annotation in circBase (http://www.circbase.org/). To observe cellular localization of the LNMAC, we conducted a FISH assay for nuclear and cytoplasmic LNMAC. Results showed that LNMAC mainly localized to the cytoplasm of SiHa and SW756 cells (Figure 2C), which was further confirmed by RT‐qPCR analysis (Figures 2D and S4A, Supporting Information). Additionally, we found that LNMAC expression was more stable than its linear mRNA counterpart after actinomycin D treatment at several indicated time points (Figures 2E and S4B, Supporting Information). Besides, results showed that LNMAC was resistant to RNase R treatment, whereas its linear counterpart was easily digested (Figure S4C,D, Supporting Information). Since circular RNAs do not possess a 3´‐poly adenylated tail, we used random primers or oligo dT primers to synthesize reverse transcript products from SiHa and SW756 cells. Results showed that LNMAC was only detectable in cDNA generated using random primers (Figure S4E, Supporting Information).

### 2.3 LNMAC Overexpression Correlates with Lymphatic Metastasis of CSCC Patients

To validate LNMAC expression in CSCC tissues, we enrolled a large clinical cohort consisting of cervical tissue samples from 201 CSCC cases and 99 healthy controls. LNMAC levels, probed by quantitative reverse transcription PCR (RT‒qPCR), were significantly higher in tumor samples than in non‐tumor tissues (Figure 2F). We further stratified CSCC samples and showed that LNMAC had increased expression in the tumors with lymphvascular space invasion (LVSI) relative to tumors without LVSI (Figure 2G). Moreover, the relationship between LNMAC expression and the clinicopathological characteristics of patients with CSCC was investigated (Table S4, Supporting Information). We found that higher expression levels of LNMAC in primary CSCC samples were associated with lymphovascular space invasion (*P* < 0.0001) and lymph node metastasis (*P* < 0.0001). Notably, Kaplan‐Meier survival curves and log‐rank test analyses showed that high abundance of LNMAC in CSCC samples was associated with shorter overall survival (OS) (Figure 2H). Furthermore, statistical analysis revealed that LNM‐positive CSCC tissues possessed higher LNMAC expression than LNM‐negative CSCC tissues in our large clinical cohort (Figure 2I). Importantly, LNMAC was overexpressed in high‐LNR (Lymph node ratio) CSCC tissues compared to low‐LNR CSCC tissues (Figure 2J). Furthermore, we observed a progressive gain of LNMAC expression in CSCC tissues with LNM ≥ 4 (Figure 2K). Consistently, ISH revealed that LNMAC was significantly overexpressed in CSCC with LN metastasis compared with those without LN metastasis (Figure 2L). LNMAC overexpression was accompanied by an increased density of D2‐40 (podoplanin)‐marked microlymphatic vessels in both intratumoral and peritumoral regions (Figure 2M–P), indicating that LNMAC plays an important role in LN metastasis and lymphangiogenesis in CSCC. Collectively, these results suggest that LNMAC is closely related to LN metastasis of CSCC.

### 2.4 LNMAC Promotes CSCC Lymph Node Metastasis In Vivo

**Figure 3:**
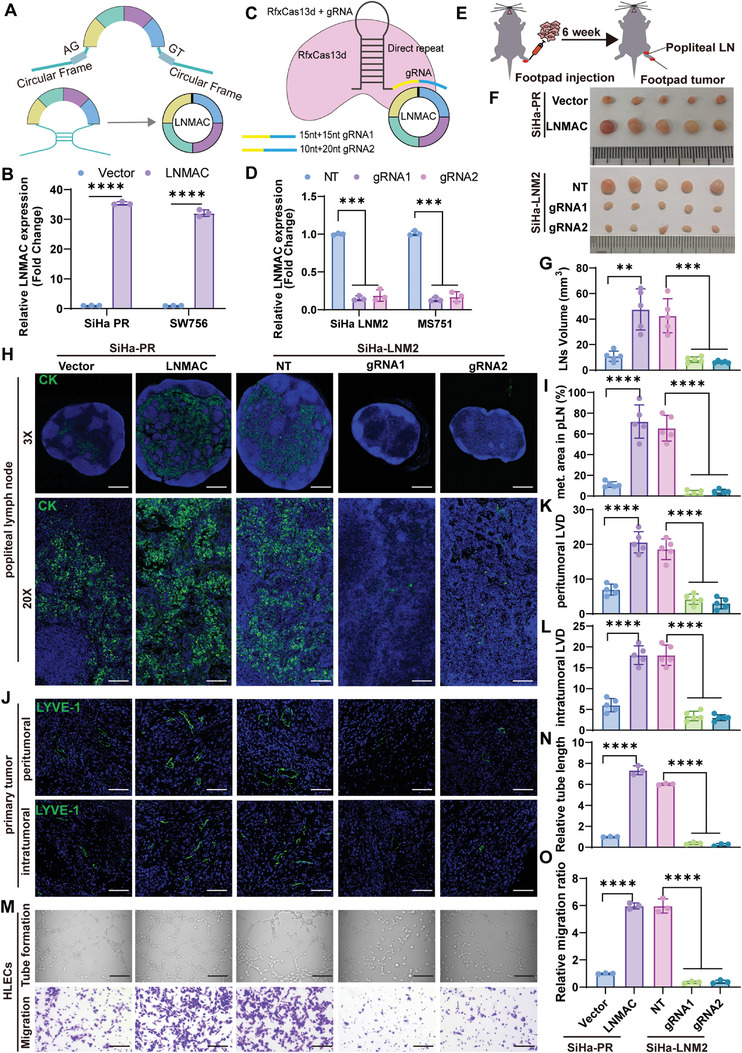
LNMAC promotes CSCC lymph node metastasis in vivo. A,B) The schematic illustration of LNMAC expression vector, and the expression levels of LNMAC in SiHa and SW756 cells stably transfected with LNMAC or corresponding negative control were detected by RT‐qPCR (*n* = 3). C,D) Schematic of circRNA knockdown using RfxCas13d‐BSJ‐gRNA system. Two BSJ‐gRNAs targeting the BSJ site were designed for LNMAC. The knockdown efficiency for each gRNA of circVPRBP was evaluated by RT‐qPCR (*n* = 3). E,F) The schematic illustration of in vivo nude mouse LN metastasis model of CSCC, and the representative images of popliteal LNs in different groups (*n* = 5). G) LNs volumes in different groups (*n* = 5). H,I) Immunofluorescent staining for pan‐cytokeratin of popliteal LNs in different groups, and the metastatic area of popliteal LNs in each group (*n* = 5). J–L) Representative images of immunofluorescent staining and quantification of lymphatic vascular density in peritumoral and intratumoral regions (*n* = 5). M–O) Tube formation and transwell assays of HLECs treated with conditioned medium from LNMAC‐ overexpressing or silencing CSCC cells (*n* = 3). Each experiment was performed at least three times independently. **P < 0.01; ***P < 0.001; ****P < 0.0001.

We used a circRNA‐expression system and RfxCas13‐gRNA mediated circRNA knockdown system to successfully overexpress and silence LNMAC expression in SiHa‐PR/LNM2 and SW756 cells (**Figure** **3**A–D). To investigate the impact of LNMAC on nodal metastasis of CSCC, an in vivo nude mouse LN metastasis model (Figure 3E), which simulates the directional drainage and metastasis of lymph nodes of cervical cancer, was employed. The cervical cancer cells were implanted into the footpads of nude mice and the popliteal lymph nodes were removed and analyzed. Strikingly, LNMAC overexpression notably promoted LN metastasis of SiHa‐PR cells. Conversely, silencing LNMAC suppressed the ability of the highly metastatic SiHa‐LNM2 cells to metastasize to the LNs (Figure 3F). The volumes of the popliteal LNs were larger in the LNMAC tumor group than in the control group, whereas the volumes of the LNs were significantly smaller in the gRNA‐LNMAC group than in the control group (Figure 3G). Immunostaining of pan‐cytokeratin confirmed that forced expression of LNMAC significantly increased the lymphatic metastatic ability of parental SiHa cells and ablation of LNMAC inhibited LN metastasis of SiHa‐LNM2 cells (Figure 3H,I). Moreover, through orthotopic pelvic lymph node metastatic model, the tumor cells were implanted into the uterus of nude mice and the pelvic lymph nodes were removed and analyzed. Reconciling with our previous results, ablation of LNMAC abrogated the capability of the highly metastatic SiHa‐LNM2 cells to metastasize to the pelvic LNs (Figure S5A,B, Supporting Information). Both the volumes and metastatic area of the pelvic LNs were great smaller in the LNMAC‐gRNA1 tumor group than in the control group (Figure S5C,D, Supporting Information). Together, these findings suggest that LNMAC augments LN metastasis of CSCC in vivo.

### 2.5 LNMAC Elicits Lymphangiogenesis in CSCC

Lymphangiogenesis is the rate‐limiting step of tumor LN metastasis, hence blocking lymphangiogenic signaling pathways might be a useful therapeutic strategy to restrict nodal metastasis.^[^ ^22^ ^]^ Since LNMAC was positively correlated with LVSI and lymphatic vessel density in primary tumors, it seemed plausible that LNMAC could influence lymphangiogenesis in CSCC. To explore this possibility, we conducted immunofluorescence analysis using a lymphatic vessel marker, LYVE‐1, to quantify intratumoral and peritumoral lymphatic vessels in primary footpad and orthotopic tumors. Interestingly, LYVE‐1 positive vessels significantly increased in mice bearing LNMAC overexpression cells and declined in mice inoculated with gRNA‐LNMAC cells in both intratumoral and peritumoral regions (Figures 3J–L and S5E–G, Supporting Information). This was consistent with previous screening findings, indicating that LNMAC elicits lymphangiogenesis in vivo.

To further elaborate the functional impact of LNMAC on lymphangiogenesis in vitro, the culture media (CM) derived from equal numbers of LNMAC‐overexpressing, LNMAC‐knockdown CSCC cells and control CSCC cells were harvested to treat human lymphatic endothelial cells (HLECs). Expectedly, conditioned media derived from LNMAC overexpressing SiHa‐PR and SW756 cells obviously enhanced tube formation and migration of HLECs compared to controls. Conversely, the tube formation and migration of HLECs were markedly impaired after incubation with CM from LNMAC‐knockdown SiHa‐LNM2 and MS751 cells (Figures 3M–O, and S6A–F, Supporting Information), suggesting that LNMAC significantly enhances the lymphangiogenesis of CSCC.

### 2.6 LNMAC Interacts with HDAC1 to Promote Lymphangiogenesis and Nodal Metastasis

**Figure 4:**
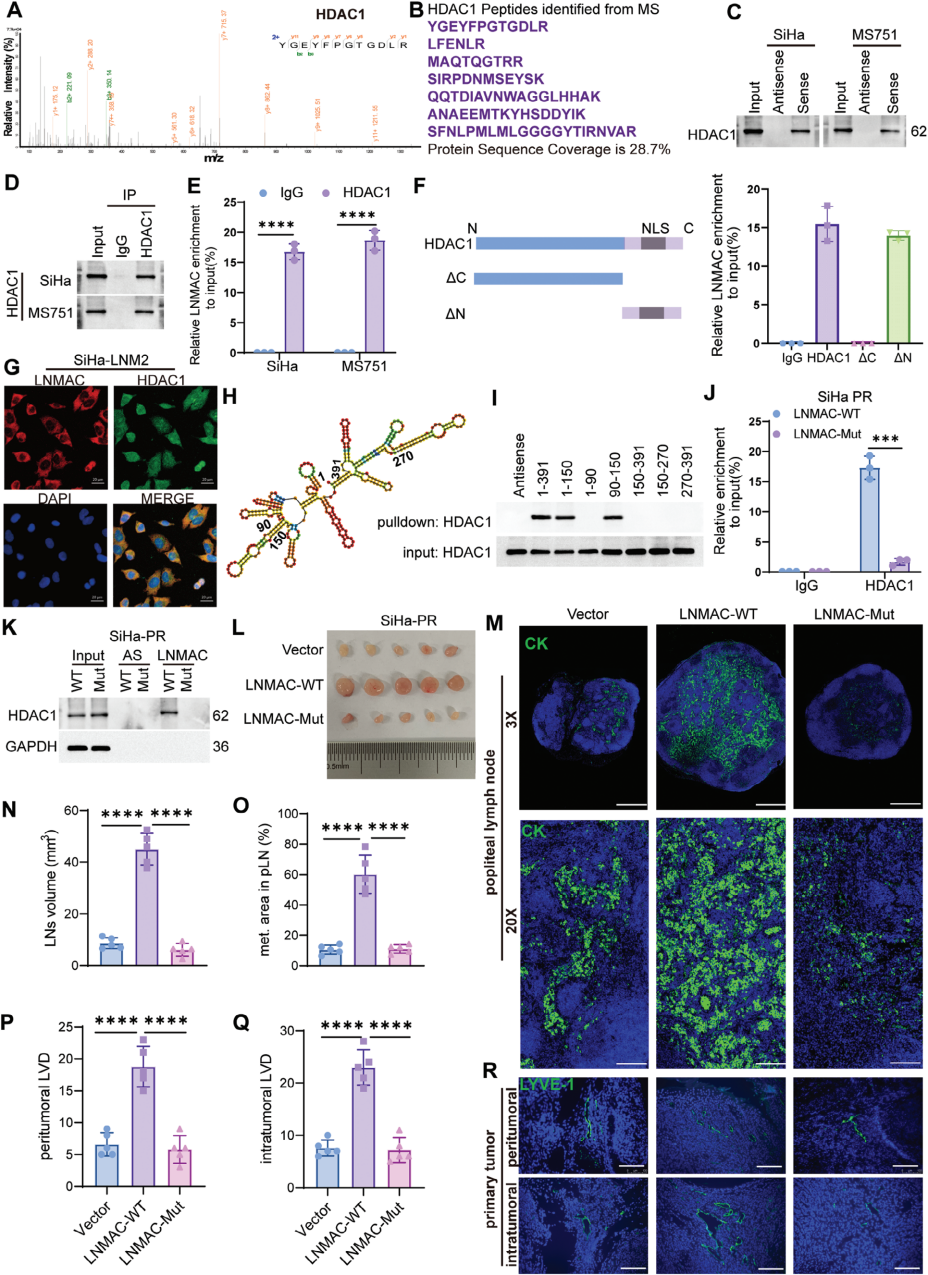
LNMAC interacts with HDAC1 to promote lymphangiogenesis and nodal metastasis. A,B) Mass spectrometry analysis of LNMAC‐binding proteins after RNA pull‐down assay. C) Western blotting of the interaction between LNMAC and HDAC1. D,E) Binding of LNMAC to HDAC1 in SiHa and SW756 cells was detected by RIP assay. F) Schematic illustration of full‐length and deleted HDAC1 proteins (left). RIP assay showed that the full‐length HDAC1 and the ΔN‐HDAC1, but not the ΔC‐HDAC1 was interacted with LNMAC (right). G) Fluorescence colocalization assay showed that LNMAC and HDAC1 protein colocalize in the cytoplasm of SiHa‐LNM2 cells. H) The predicted secondary structure of LNMAC using the RNAfold Web Server based on the minimum free energy. Color scales indicated the confidence of predictions for each base. I) RNA pulldown assay showed HDAC1 was pulled down by biotin‐labeled LNMAC of different lengths. J) RIP and K) pull‐down assay showed the interaction between HDAC1 and LNMAC‐WT, rather than LNMAC‐Mut in SiHa‐PR cells. L) Representative images of popliteal LNs and immunofluorescent staining of CK in different groups (*n* = 5). N,O) Volume and metastatic area of popliteal LNs in each group (*n* = 5). P,Q) Representative images of LYVE1 positive lymphatic vessels and the lymphatic vascular density in peri‐ and intratumoral regions in different groups (*n* = 5). Each experiment was performed at least three times independently. ***P < 0.001; ****P < 0.0001.

To further investigate the molecular mechanism underlying LNMAC‐induced lymphatic metastasis, we performed RNA pull‐down assays. Biotinylated sense (S) or antisense (AS) DNA oligomers spanning the LNMAC back‐splicing junction site, were introduced into cells. The LNMAC‐bound proteins were then subjected to mass spectroscopy analysis. We found that HDAC1 was identified as the most abundant (coverage = 28.7%) protein harboring the highest score among the top‐ranked putative binding proteins of LNMAC (**Figures** **4**A,B and S7, Supporting Information). The association between LNMAC and HDAC1 was validated using sense probes of LNMAC (Figure 4C). An RNA immunoprecipitation (RIP) assay further confirmed the specific interaction of HDAC1 with LNMAC (Figure 4D,E). Moreover, we constructed an C‐terminus residue deletion HDAC1‐Flag plasmid (ΔC‐HDAC, amino acids 1‐322) and a N‐terminus residue deletion HDAC1‐Flag plasmid (ΔN‐HDAC1, amino acids 323‐482) and probed their LNMAC‐binding abilities. Both the full‐length HDAC1 and ΔN‐HDAC1 interacted with LNMAC, but the ΔC‐HDAC1 construct did not (Figure 4F), indicating that LNMAC binds to C‐terminus residues of HDAC1 protein. Moreover, fluorescence colocalization studies showed that LNMAC and HDAC1 colocalize in the cytoplasm of SiHa‐LNM2 cells (Figure 4G). Furthermore, various truncated constructs of LNMAC molecules and RNA pull‐down assays revealed that HDAC1 strongly interacted with a 90‐150 nt motif of LNMAC (Figure 4H,I). To determine whether the 90–150 nt motif contributes to HDAC1 association, we constructed a mutant LNMAC (90‐150 nt), and the endogenous interaction between HDAC1 and LNMAC‐WT, rather than LNMAC‐Mut was validated by both RIP (Figures 4J and S8A, Supporting Information) and pull‐down assays (Figures 4K and S8B, Supporting Information). These supported a direct interaction between LNMAC and HDAC1 via the 90‐150 nt region.

To ascertain whether direct interaction of LNMAC and HDAC1 contributes to the lymphatic metastatic effect of LNMAC, SiHa‐PR cells with enforced expression of LNMAC‐WT or equivalent LNMAC‐Mut variants, were employed to evaluate their nodal metastasis abilities. As expected, enforced LNMAC‐Mut expression failed to promote lymphatic metastasis based on findings related to the volume of popliteal LNs and metastatic area of SiHa‐PR cells (Figure 4M–O). Similar phenomena were also observed regarding lymphangiogenesis. Wild type LNMAC promoted LYVE1 positive lymphatic vessel formation in both peri‐ and intratumoral regions when compared to the HDAC1 interaction‐deficient LNMAC mutant (Figure 4P–R). Furthermore, in vitro assays indicated that the culture media retrieved from mutant LNMAC cells could not accelerate tube formation (Figure S8C,D, Supporting Information) and migration of HLEC cells (Figure S8E,F, Supporting Information). These findings further supported the contribution of LNMAC and HDAC1 interaction on CSCC lymphangiogenesis and lymphatic metastasis.

### 2.7 LNMAC Inhibits Importin α6/8‐Mediated HDAC1 Nuclear Translocation and Epigenetically Activates FGF2 Expression

**Figure 5:**
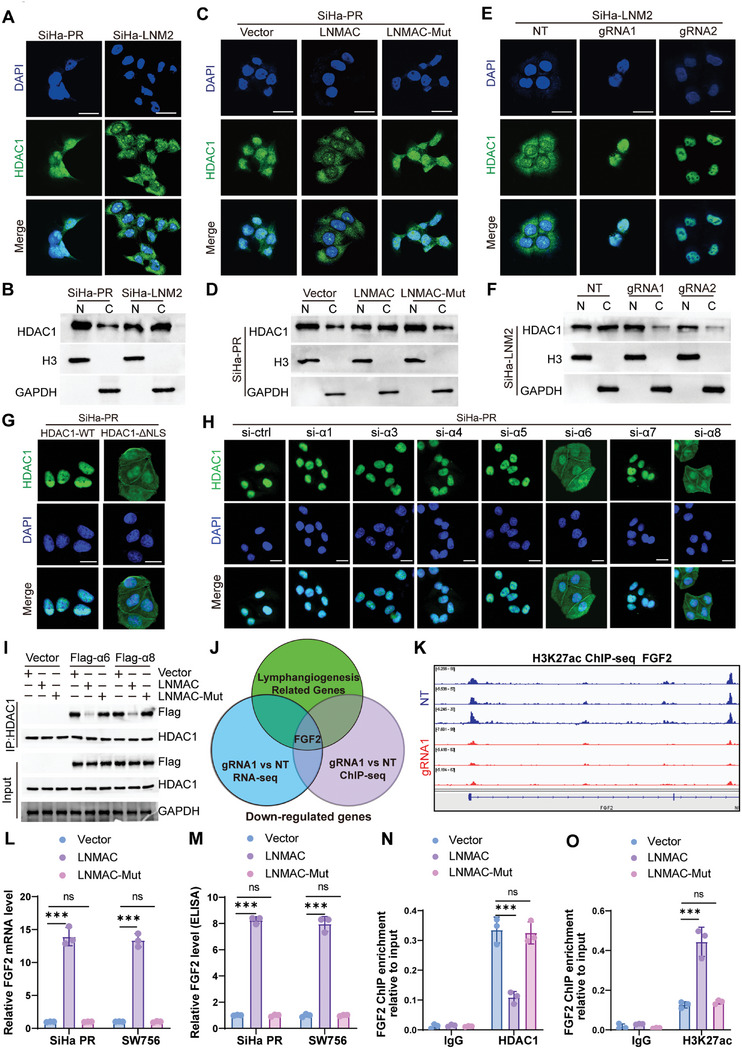
LNMAC inhibits importin α6/8‐mediated HDAC1 nuclear translocation and epigenetically activates FGF2 expression. A–H) Immunofluorescent staining of HDAC1 and western blotting showed the different location of HDAC1 in the indicated groups. I) Coimmunoprecipitation experiment confirmed that ectopic expression of wild‐type LNMAC, but not LNMAC mutant, significantly reduced binding of FLAG‐tagged importin α6 and α8 with HDAC1. J) Venn diagram demonstrating the overlapping genes in three groups. K) Browser image of H3K27ac ChIP‐seq results at locus FGF2. L,M) RT‐qPCR and ELISA assay of FGF2 expression in different groups (*n* = 3). N,O) Quantification of immunoprecipitated protein expression normalized by respective input in different groups (*n* = 3). Each experiment was performed at least three times independently. ns, no significance; ***P < 0.001.

Given that the LNMAC interacts with the C terminus of HDAC1, which contains a nuclear localization signal (NLS), and we previously found that LNMAC and HDAC1 colocalize in the cytoplasm of SiHa‐LNM2 cells; it seemed likely that LNMAC interacts with the C terminus of HDAC1, blocking the NLS and suppressing the nuclear localization of HDAC1. To explore this possibility, we first quantified HDAC1 mRNA and protein expression after gain‐ and loss‐of LNMAC in CSCC cells. No significant alteration in HDAC1 expression was observed (Figure S9A–C, Supporting Information). Of note, immunofluorescent staining showed that SiHa‐LNM2 cells with relative higher LNMAC expression had significantly reduced HDAC1 nuclear localization when compared to SiHa‐PR cells (**Figure** **5**A). This was confirmed by nuclear and cytoplasmic protein fraction assays (Figure 5B). Besides, enforced expression of wild type LNMAC, rather than mutant LNMAC, decreased HDAC1 nuclear localization in SiHa‐PR cells (Figure 5C,D), whereas ablation of LNMAC augmented HDAC1 nuclear localization in SiHa‐LNM2 cells (Figure 5E,F). These findings suggest that LNMAC may abrogate HDAC1 nuclear translocation in CSCC cells.

HDAC1 contains a NLS on the C terminus, and deletion of the NLS prevented nuclear accumulation of HDAC1 in SiHa‐PR cells (Figure 5G). Importins are proteins that mediate transport of proteins through the nuclear pore. There are seven importin α proteins to recognize cargo protein NLSs in cells. Through siRNA screening, Importin α6 and importin α8 appeared to contribute to nuclear localization of HDAC1 (Figure 5H). Given these findings, we hypothesized that LNMAC regulates nuclear translocation of HDAC1 by affecting the interaction between HDAC1 and Importin α6 and importin α8. Coimmunoprecipitation experiments confirmed that ectopic expression of wild‐type LNMAC, but not LNMAC mutant, significantly reduced binding of FLAG‐tagged importin α6 with HDAC1 and also reduced the association of HDAC1 with importin α8 in SiHa‐PR cells (Figure 5I).

HDAC1 plays a central role in the epigenetic regulation of gene expression.^[^ ^23^, ^24^ ^]^ To investigate the epigenetic target underlying LNMAC/HDAC1‐regulation of lymphangiogenesis and lymphatic metastasis, we subjected control and gRNA‐LNMAC SiHa‐LNM2 cells to ChIP‐seq and RNA‐seq analyses. We overlapped mutually down‐regulated genes in both anti‐H3K27ac ChIP‐seq and RNA‐seq upon LNMAC knockdown, with lymphangiogenesis‐related genes. FGF2 was identified as the only target that might mediate LNMAC induced lymphangiogenesis in CSCC (Figure 5J,K). In addition, forced expression of the wild type LNMAC, rather than mutant LNMAC, induced FGF2 expression and secretion (Figures 5L,M andS9D,E, Supporting Information). Moreover, ChIP‐qPCR assays confirmed that HDAC1 occupied the promoters of FGF2, which was significantly reduced by LNMAC upregulation in SiHa‐PR cells (Figure 5N), followed by significantly increased histone acetylation level at the transcriptional activation sites (H3K27) of FGF2 promoter (Figure 5O). Conversely, LNMAC knockdown remarkably repressed FGF2 expression (Figure S9F–H, Supporting Information), enhancing the binding of HDAC1 to the FGF2 promoter, and decreasing acetylation of H3K27ac at FGF2 promoter (Figure S9I,J, Supporting Information). The role of HDAC1 in the regulation of FGF2 expression was also verified by treating cells with trichostatin A (TSA), a HDAC inhibitor.^[^ ^25^ ^]^ TSA treatment upregulated FGF2 expression in SiHa‐PR cells in a dose‐ and time‐dependent manner (Figure S9K,L, Supporting Information), rescuing FGF2 expression induced by ablation of LNMAC (Figure S9M, Supporting Information). To further clarify whether LNMAC activated FGF2 expression via induction of HDAC1 nuclear translocation deficiency, we generated a ΔNLS‐HDAC1 plasmid which lacked the NLS and was unable to translocate to the nucleus. Enforced expression of full length HDAC1, rather than ΔNLS‐HDAC1, abrogated the LNMAC elicited FGF2 expression in SiHa‐PR and SW756 cells (Figure S10A,B, Supporting Information). Similarly, culture media from cells with ectopic expression of full length HDAC inhibited LNMAC‐enhanced tube formation and migration of HLEC cells, whereas ΔNLS‐HDAC1 showed no effect (Figure S10C–F, Supporting Information). These findings indicate that LNMAC promotes lymphangiogenesis and FGF2 expression through cytoplastic retention of HDAC1.

### 2.8 FGF2 Is Required for LNMAC‐Induced Lymphangiogenesis and Lymphatic Metastasis

**Figure 6:**
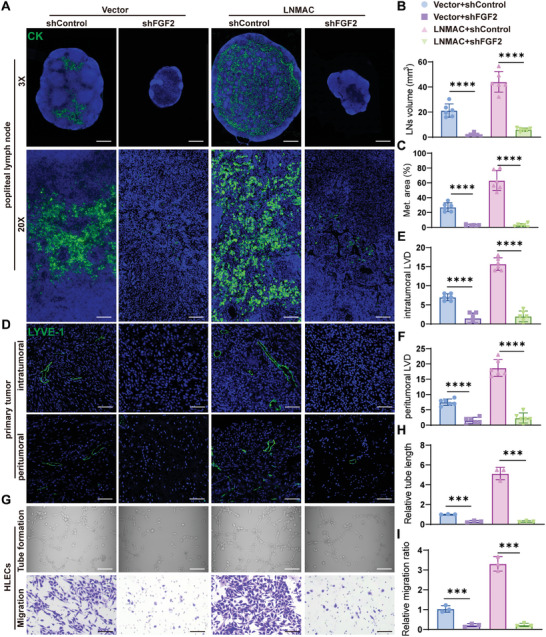
FGF2 is required for LNMAC‐induced lymphangiogenesis and lymphatic metastasis. A) Representative images of immunofluorescent staining for pan‐cytokeratin. B,C) Volume and metastatic area of popliteal LNs in different groups (*n* = 6). D–F) Representative images of LYVE‐1 positive lymphatic vessels and the lymphatic vascular density in intra‐ and peritumoral regions of different group (*n* = 6). G) Representative images of tube formation and transwell assays of HLECs treated with conditioned medium from CSCC cells. H,I) Statistical analysis of relative length of tubes and migration ratio in each group (*n* = 3). Each experiment was performed at least three times independently. ***P < 0.001; ****P < 0.0001.

Accumulating evidence has shown that FGF2 signaling plays important roles in tumor lymphangiogenesis and lymph node metastasis,^[^ ^26^, ^27^, ^28^ ^]^ and may serve as a promising therapeutic target for lymphatic metastatic cancer. Thus, we investigated whether loss of FGF2 could suppress LNMAC‐induced nodal metastasis. FGF2 silencing showed more inhibitory effects on popliteal lymph node metastasis of LNMAC‐transduced SiHa‐PR cells relative to control cells (**Figures** **6**A–C and S11A, Supporting Information). Ablation of FGF2 also repressed the intratumoral and peritumoral lymphangiogenesis observed in the LNMAC‐expressing mice (Figure 6D–F). Concordant with the previous findings, knockdown of FGF2 significantly inhibited LNMAC‐induced HLECs tube formation and migration (Figure 6G–I), indicating that FGF2 is required for LNMAC‐induced lymphangiogenesis and lymph node metastasis in CSCC.

### 2.9 Targeting FGF2‐Induced Lymphangiogenesis Is an Effective Treatment of CSCC Lymph Node Metastasis

**Figure 7:**
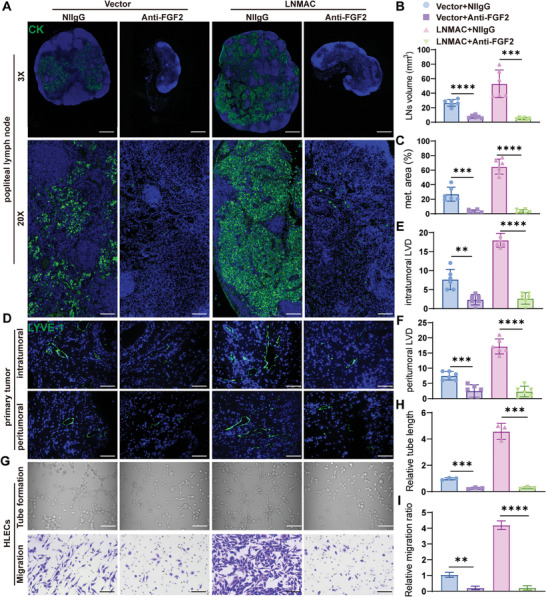
Targeting FGF2‐induced lymphangiogenesis is an effective treatment of CSCC lymph node metastasis. A) Representative images of immunofluorescent staining of pan‐cytokeratin. B,C) Volume and metastatic area of popliteal LNs in different groups (*n* = 6). D–F) Representative images of LYVE‐1 positive lymphatic vessels and the lymphatic vascular density in intra‐ and peritumoral regions (*n* = 6). G–I) Tube formation and transwell assays of HLECs treated with conditioned medium from CSCC cells of different groups (*n* = 3). Each experiment was performed at least three times independently. **P < 0.01; ***P < 0.001; ****P < 0.0001.

**Figure 8:**
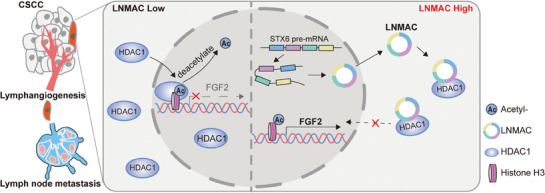
A schematic diagram of the mechanism. LNMAC could bind to HDAC1 and inhibit its nuclear translocation, resulting in elevated FGF2‐mediated lymphangiogenesis and lymph node metastasis in CSCC.

A FGF2 trap molecule (FP‐1039) has been tested in a clinical trial for patients with malignant solid tumors.^[^ ^15^, ^29^ ^]^ Recently, a new anti‐FGF2 monoclonal antibody (mAb), 3F12E7, was used in a B16‐F10 melanoma model and could reduce the number and size of metastatic foci in lungs.^[^ ^30^ ^]^ We evaluated whether the therapeutic use of an anti‐FGF2 monoclonal antibody (3F12E7) could inhibit LNMAC‐induced lymph node metastasis. Reconciling with the effect of FGF2 ablation, treatment with the neutralizing antibody against FGF2 (3F12E7) significantly reduced popliteal lymph node volume and LNMAC‐transduced tumor burden (**Figures** **7**A–C and S11B, Supporting Information). It also led to a remarkable reduction in both intratumoral and peritumoral lymphatic vessel densities (Figure 7D–F). Moreover, the 3F12E7 antibody significantly repressed LNMAC‐induced HELCs tube formation and motility (Figure 7G–I), suggesting that deactivation of FGF2 signaling may inhibit LNMAC‐mediated lymphangiogenesis in vitro. We also confirmed that using anti‐FGF2 monoclonal antibody (3F12E7) could inhibit lymphangiogenesis and lymph node metastasis in the orthotopic lymph node metastasis model. The volumes and the metastatic area of the pelvic LNs were significantly smaller in the anti‐FGF2 group than in the control group (Figure S11C–F, Supporting Information). Meanwhile, anti‐FGF2 monoclonal antibody (3F12E7) could decrease newborn lymphatic vessels in both intratumoral and peritumoral regions of orthotopic uterine tumors (Figure S11G–I, Supporting Information). Taken together, these results demonstrate that targeting FGF2‐induced lymphangiogenesis is an effective treatment of CSCC lymph node metastasis(**Figure** **8**).

## 3 Discussion

In the present study, we first analyzed circRNA‐induced epigenetic regulation of FGF2 and lymphatic metastasis in CSCC. We showed that LNMAC was markedly upregulated in lymph node metastatic CSCC cells and tissues. LNMAC overexpression epigenetically promoted intratumoral and peritumoral lymphangiogenesis of CSCC via direct interaction with HDAC1, a core component of the histone deacetylase complex. LNMAC activated FGF2‐mediated lymphangiogenesis through decoying and decreasing HDAC1 nuclear translocation. Suppression of lymphangiogenesis using a FGF2 monoclonal antibody significantly inhibited nodal metastasis of LNMAC‐overexpressing CSCC cells in vivo. These results provide mechanistic and translational insights into the lymphatic metastasis of CSCC and suggest that the inhibition of lymphangiogenesis using a FGF2 antibody may serve as a potential targeted treatment option for patients with LNMAC‐overexpressing CSCC.

Lymphangiogenesis is pivotal for lymph node metastasis and confers a poor prognosis to CSCC patients, who lack appropriate clinical therapies.^[^ ^31^, ^32^ ^]^ Cancer cells secrets lymphangiogenic growth factors to induce new lymphatic vessel formation and assist tumor nodal spread.^[^ ^33^, ^34^ ^]^ Thus, a better understanding of the molecular mechanisms underlying lymphangiogenesis may help provide effective clinical intervention for CSCC.

Herein, we investigated the critical role of circRNA in facilitating tumor lymphangiogenesis, thus providing new insight into the interaction of circRNA and lymphatic metastasis. Through in vivo lymphatic metastasis and lymphangiogenesis activation screening, we found LNMAC overexpression notably promoted lymphangiogenesis and nodal metastasis in a xenograft popliteal lymph node metastasis model. Conversely, RfxCas13d induced ablation of LNMAC suppressed lymphangiogenesis in vitro and in vivo. Therefore, it seems plausible that LNMAC supplies a favorable condition for lymphangiogenesis and nodal metastasis in CSCC.

It is becoming evident that the effects of FGF2 on tumor lymphangiogenesis and progression are far more important than previously assumed.^[^ ^13^, ^35^ ^]^ Generally, FGF2 positively correlates with regional LN metastasis in multiple human malignancies, including CSCC.^[^ ^14^ ^]^ FGF2 plays a role in the lymphangiogenic switch by triggering the activation of lymphatic endothelial cells in cooperation with VEGFs through FGFR1/VEGFR3‐dependent pathways,^[^ ^26^ ^]^ necessary to disrupt the endothelial lymphatic barrier and facilitate lymphatic invasion. FGF2 has also been shown to interact with LYVE‐1 which participates in FGF2 internalization, ultimately resulting in significantly enhanced lymph node metastasis and a clinical treatment dilemma.^[^ ^36^ ^]^ It has been reported that using anti‐FGF2 antibodies or FGF trap neutralizing antibodies to block FGF2‐mediated signaling pathways, could suppress the metastatic spread of human malignancies.^[^ ^15^, ^29^, ^30^ ^]^ Moreover, FP‐1039 (GSK3052230), a FGF2 trap molecule, has been tested in a clinical phase IB trial for patients with malignant solid tumors (ClinicalTrials.gov NCT01868022).^[^ ^15^ ^]^ Therefore, exploring the underlying molecular mechanism by which FGF2 is overexpressed in CSCC may chart the course for effective anti‐FGF2 treatment. In the present study, we have identified a circRNA, LNMAC, which augments lymphangiogenesis and lymphatic metastasis by epigenetically activating FGF2 expression in CSCC. Moreover, we observed that genetic knockdown FGF2 or a neutralizing monoclonal antibody (3F12E7) had effective anti‐lymphangiogenic functions in LNMAC‐overexpressing CSCC tumors. Therefore, our findings reveal a molecular mechanism for the lymphangiogenesis and lymphatic metastasis of CSCC and indicate that LNMAC might serve as a potential biomarker for clinical treatment in nodal metastatic CSCC.

Epigenetic regulation of gene expression has been implicated in tumor metastasis.^[^ ^37^, ^38^, ^39^ ^]^ HDAC1 plays a critical role in the epigenetic regulation of gene expression and the role of LNMAC in mediating HDAC1 cytoplasmic retention is an interesting paradigm. HDAC1 has a NLS and is mainly located in the nucleus.^[^ ^40^ ^]^ Herein, we propose a model in which LNMAC sequestrates HDAC1 in the cytoplasm, increasing the transcription of FGF2 and thus promoting CSCC lymphangiogenesis and lymphatic metastasis. These findings broadened our understanding of the molecular mechanisms underlying HDAC1 function. Multiple studies have recently reported that HDAC1 has become a promising anticancer target since HDAC inhibitors can induce tumor cell apoptosis and inhibit angiogenesis.^[^ ^41^ ^]^ Therefore, inhibition of HDAC signaling may result in broad disturbances in normal cellular functions. Currently, a series of HDAC inhibitors are being tested clinically to treat relapsing multiple myeloma and cutaneous and peripheral T‐cell lymphomas.^[^ ^42^ ^]^ However, the response to HDAC inhibitors in solid tumor treatment has been disappointing. Phase II trials using HDAC inhibitors against solid tumors revealed that only few patients reached complete remission. Of note, HDAC inhibitors can induce serious adverse effects including anemia, pulmonary embolism, leukopenia, and deep vein thrombosis, raising concerns regarding its therapeutic use. Previous literature reported that the HPV E6/E7 protein could activate the promoters of OCT4 and HIF‐1A by sequestering HDAC1 from the repressor complex to influence carcinogenesis.^[^ ^43^, ^44^ ^]^ Concordant with previous studies, we also found that HDAC1 is a suppressor of FGF2. The HDAC inhibitor, TSA, increases the expression of FGF2, suggesting that HDAC1 is a potent suppressor of lymphangiogenesis. Therefore, caution should be exercised when using HDAC inhibitors as anticancer drugs in tumors prone to lymphatic metastasis, especially CSCC.

In summary, our present work uncovers the mechanism underlying overexpression of LNMAC and promotion of CSCC lymphatic metastasis by epigenetically inducing FGF2‐associated lymphangiogenesis (Figure 8). These results provide a therapeutic basis for targeting FGF2‐induced lymphangiogenesis as an effective treatment of cancer lymph node metastasis. Based on our work, it would be interesting in the future to design clinical trials involving combination chemoradiotherapy/anti‐FGF2 agents for the treatment of advanced CSCC in patients with high LNMAC expression.

## 4 Experimental Section

### Clinical Specimens

CSCC tissues were obtained from patients who underwent radical hysterectomy and lymphadenectomy between 2014 and 2017 at the First Affiliated Hospital of Sun Yat‐sen University (Guangzhou, China). None of the enrolled CSCC patients had radiotherapy or chemotherapy prior to surgery and were at stages Ia2 to IIa2 with regular follow‐up date. This study was approved by the Ethical Review Committee of the First Affiliated Hospital of Sun Yat‐sen University (approved number: IIT‐2022‐205). Patient studies were conducted in accordance with the Declaration of Helsinki. All human tumor tissues were obtained with written informed consent from patients or their guardians prior to participation in the study.

### Cell Culture

Human CSCC cell lines (SiHa, SW756, MS751) were purchased from the American Type Culture Collection (ATCC, USA). Human lymphatic endothelial cells (HLECs) were obtained from ScienCell Research Laboratories and cultured in the ECM (ScienCell, CA). All CSCC cell lines were cultured in DMEM with 10% fetal bovine serum (Gibco, USA) and 0.5% penicillin/streptomycin (Gibco, China). Cells were cultured in a humid atmosphere with 5% CO_2_ at 37 °C. In 2022, all of the cell lines used were tested for authenticity by short tandem repeat (STR) genotyping; the cell lines were also screened for mycoplasma contamination (e‐Myco Mycoplasma PCR Detection Kit; iNtRON).

### Generation of the Lymph Node Metastasis‐Prone CSCC Cells

The metastatic pLN from the implantation of SiHa‐mCherry cells into footpad mouse model was harvested and digested, and the tumor cells were selected in vitro by addition of puromycin (2 µg mL^−1^). Then, the expanded tumor cells were reimplanted to the mice footpad. After 2 rounds of in vivo selection, highly LN metastatic SiHa subpopulation was established, named as “SiHa‐LNM2 cells.” The “parental SiHa cells” we established in the cultured cells that were isolated from primary implanted primary tumor of the mice.

### Mice Models

All animal experiments were approved by the Sun Yat‐sen University Institutional Animal Care and Use Committee (approved number: SYSU‐IACUC‐2022‐001296). Female BALB/c nude mice (4‐6 weeks of age, 18‐22 g) were purchased from the Experimental Animal Center of Sun Yat‐sen University and raised under SPF conditions. For footpad and orthotopic implantation model, CSCC cells (2 × 10^6^ / 50 µL per mouse) were implanted subcutaneously into the footpad region or the uterus of nude mice. Six weeks later, popliteal or pelvic LNs were dissected for further experiments and analyses. For the FGF2‐treatment assays, a nonimmune control IgG (50 ng mL^−1^ in HBSS) or neutralizing antibody against FGF2 (3F12E7, 50 ng mL^−1^ in HBSS) was intravenously injected. At the experimental endpoint, nude mice were anesthetized and sacrificed; their primary tumors and popliteal/pelvic LNs were enucleated and embedded in paraffin for immunohistochemistry (IHC) or immunofluorescence staining. The lymph node volumes were calculated using the following formula: Volume(mm^3^) = (length[mm])×(width[mm])^2^×0.52. The antibodies used in this study were listed in Table S1 (Supporting Information). Simple randomization was used to allocate mice into different groups and no blinding was done.

### CircRNA In Situ Hybridization (ISH) and Fluorescence In Situ Hybridization (FISH)

For FISH assay, Cy3‐labeled LNMAC probe was designed and synthesized by Geneseed (Guangzhou, China). Following manufacturer's instructions, a fluorescent in situ hybridization kit (RiboBio, Guangzhou, China) were used for hybridizations. All images were captured using a laser scanning confocal microscope (TCS SP2 AOBS). For circRNA ISH, a biotinylated ISH probe was designed by Synbio Tech (Suzhou, China) for hybridization with LNMAC and signals from the hybridized probes were detected. Sequences of FISH and ISH probes used in this study were shown in Table S2 (Supporting Information).

### Plasmid Construction and Retroviral Infection

The full length of wild type LNMAC cDNA was cloned into the lentiviral pLC5‐Puro vector to ectopically overexpress LNMAC. The mutant LNMAC plasmid was synthesized by GENEWIZ (Suzhou, China). The full length of HDAC1 cDNA and ΔNLS‐HDAC1 sequences were cloned into the lentiviral pSin‐EF1‐Puro vector to overexpress HDAC1. For RfxCas13d‐expressed stable cell lines, p23‐NES‐RfxCas13d‐Flag vector was infected into SiHa‐LNM2 and MS751 cells by lentivirus for stable cell line generation. To construct gRNA expression vectors, DNA sequences for gRNAs were synthesized and cloned into pLKO.1‐TRC containing direct repeats of each corresponding Cas13. To construct shRNA‐expression vectors, oligos were synthesized for shRNAs and cloned them into pLKO.1‐BSD plasmids. In this study, X‐tremeGENE HP DNA transfection reagent (Roche, Germany) was used to transfer the corresponding plasmid into indicated cells following the manufacturer's instructions. pMD2.G vector (2.5 µg), psPAX2 vector (7.5 µg), and an expression vector of interest (10 µg) were conferred into LentiX‐293T cells with 80% confluence cultured in a 10 cm dish to produce lentiviral particles. 48 h later, the supernatant of LentiX‐293T cells was collected and filtered through 0.45 µm filter unit. The enriched virus precipitation by Lenti‐Concentin Virus Precipitation Solution (ExCell Bio) was suspended with PBS and frozen at −80 °C, or infected cells immediately. Cells were selected with puromycin (2 µg mL^−1^) or blasticidin (10 µg mL^−1^) in culture medium.

### RNA Pull‐Down Assay

RNA pull‐down assay was performed using a magnetic RNA‐Protein Pull‐Down Kit (Cat# 20164, Thermo Fisher Scientific) according to the manufacturer's protocol. Different truncated versions of LNMAC using the T7 promoter were amplified, and TranscriptAid T7 high‐yield transcription kit (Cat# K0441, Thermo Fisher Scientific) was used to transcribe RNA in vitro. Then, biotin labeling was performed using the RNA 3 ‘End biotinylation kit (Cat# 20160, Thermo Fisher Scientific), followed by incubation of 50 pmol of 3′‐biotinated transcribed RNA with streptavidin magnetic beads, and then incubation with cell lysate. Antisense RNAs of LNMAC were used as the negative control. Retrieved proteins were used for subsequent mass spectrometry (MS) (Fitgene Biotech, China) and Western blotting. MS analysis was conducted using a Q exactive hybrid quadrupole‐orbitrap mass spectrometer (ThermoFisher Scientific). MS data have been provided in Table S3 (Supporting Information). Protein identification was performed using MASCOT software by searching Uniprot_Aedis Aegypti.

### RNA Immunoprecipitation (RIP) and Chromatin Immunoprecipitation Assay (ChIP)

RIP assays were performed using the EZ‐Magna RIP Kit (Millipore) and ChIP experiments were performed using the EZ‐Magna ChIP A/G Kit (Millipore, Catalog # 17‐10086) at 4 °C as previously described. Briefly, for the RIP assays, 1% formaldehyde was pre‐cooling and used to cross‐linked 1 × 10^7^ CSCC cells cultured in the 15 cm dish. Then, the cell extracts were collected and incubated with an anti‐HDAC1or anti‐Flag antibody at 4 °C overnight. The next day, protein A/G Dynabeads were used to clear the RNA–protein complexes, and then the RNA molecules were then extracted from RNA–protein complexes and analyzed by qRT‐PCR. For each ChIP assay, 1 × 10^6^ cells were fixed in 1% formaldehyde at room temperature and the nucleus was isolated with nuclear lysis buffer supplemented with a protease inhibitor cocktail. Chromatin DNA was sonicated and sheared to a length between 200 and 1000 bp. The sheared chromatin was immunoprecipitated at 4 °C overnight using anti‐H3K27ac(R26244, ZENBIO) or anti‐HDAC1(10197‐1‐AP, Proteintech and ET1605‐35, HUABIO). Primers for RT‐qPCR, RIP‐qPCR and ChIP‐qPCR are listed in Table S2 (Supporting Information).

### HLECs Transwell Assay and Tube Formation Assay

For transwell assays, 30 000 cells were seeded into the upper chamber (Falcon) with culture medium but devoid of FBS, while the lower chamber was 700 µL complete medium of indicated CSCC cells. After 24 h, the cells on the lower surface of the chamber were fixed and then stained. The numbers of migrated cells were counted under microscope. For HLECs tube formation assay, 10 000 HLECs were seeded into 48‐well plates (precoated with matrigel) containing cell culture medium and incubated for 10 h. Tube formation was quantified by measuring the total length of tube structures or the number of branch sites/nodes in 3 random fields.

### RNA and Genomic DNA(gDNA) Extraction

The SteadyPure Universal RNA Extraction Kit (Accurate Biotechnology (Hunan) Co., LTD, Changsha, China) was used to extract the total RNA from cells or tissues following the manufacturer's instructions. A Fastpure Cell/Tissue DNA Isolation Mini Kit (Vazyme, China) was used to extract the genomic DNA.

### Cytoplasmic and Nuclear RNA/Protein Isolation

A PARIS Kit (Ambion, Life Technologies, USA) was used to isolate the nuclear and cytoplasmic fractions. Then, the extracted RNA or protein were analyzed to clarify the subcellular localization of LNMAC and HDAC1. GAPDH, Cdr1as, and U6 were used as positive control of cytoplasmic transcript and nuclear transcript respectively. GAPDH and H3 were used as positive control of cytoplasmic protein and nuclear protein respectively.

### Statistical Analysis

GraphPad Prism Version 9.0 was used to construct graphs and perform statistical analyses in this study. Two‐tailed Student's t test was used to analyze differences between groups. Overall survival (OS) was analyzed with the Kaplan‐Meier method and calculated by log‐rank test. Correlations were analyzed by using Pearson's correlation. Error bars represent mean ± SD. The difference was considered statistically significant with *p < 0.05, **p < 0.01, ***p < 0.001 and ****p < 0.0001.

### Ethics Statement

The studies using human tissue samples was approved by the Ethics Committee of The First Affiliated Hospital of Sun Yat‐sen University (approved number: IIT‐2022‐205). Animal experiments were approved by the Animal Ethical and Welfare Committee of Sun Yat‐sen University (approved number: SYSU‐IACUC‐2022‐001296) and were performed with the provisions of the Declaration of Helsinki of 1975.

## Conflict of Interest

The authors declare no conflict of interest.

## Author Contributions

C.Z., L.Y., W.Wen., and C.S. contributed equally to this work. C.Z., W.Wang., and S.Y. participated in the study design. C.Z., L.Y., W.Wen., and C.S. conducted the in vitro and in vivo experiments. Y.J., Y.D.L., D.X., Y.L., L.C., and X.Z. performed the data analyses, G.Y., H.J., W.Wang., and S.Y. collected the tissue specimens, C.Z., L.Y., and S.Y. wrote the manuscript. All authors have read and approved of the final manuscript.

## Supporting information

Supporting Information

## Data Availability

The data that support the findings of this study are available from the corresponding author upon reasonable request.
