# Supplementary material for: LNMAC Promotes Cervical Squamous Cell Carcinoma Lymphatic Metastasis via Epigenetic Regulation of FGF2‐Induced Lymphangiogenesis
Source: Adv Sci (Weinh). 2024 Aug 9;11(38):2404645. doi: 10.1002/advs.202404645 (PMC11481257; doi:10.1002/advs.202404645)
Supplement: Supplementary file 1 — Supporting Information [file ADVS-11-2404645-s001.pdf]

ADVANCED SCIENCE
Open Access

# Supporting Information

for *Adv. Sci.*, DOI 10.1002/advs.202404645

LNMAC Promotes Cervical Squamous Cell Carcinoma Lymphatic Metastasis via Epigenetic Regulation of FGF2-Induced Lymphangiogenesis

*Chunyu Zhang, Li Yuan, Weijia Wen, Caixia Shao, Yuandong Liao, Yan Jia, Xueyuan Zhao, Yan Liao, Dingze Xu, Linna Chen, Guofen Yang, Hongye Jiang*, Wei Wang* and Shuzhong Yao**

## Supplementary Information

Figure S1

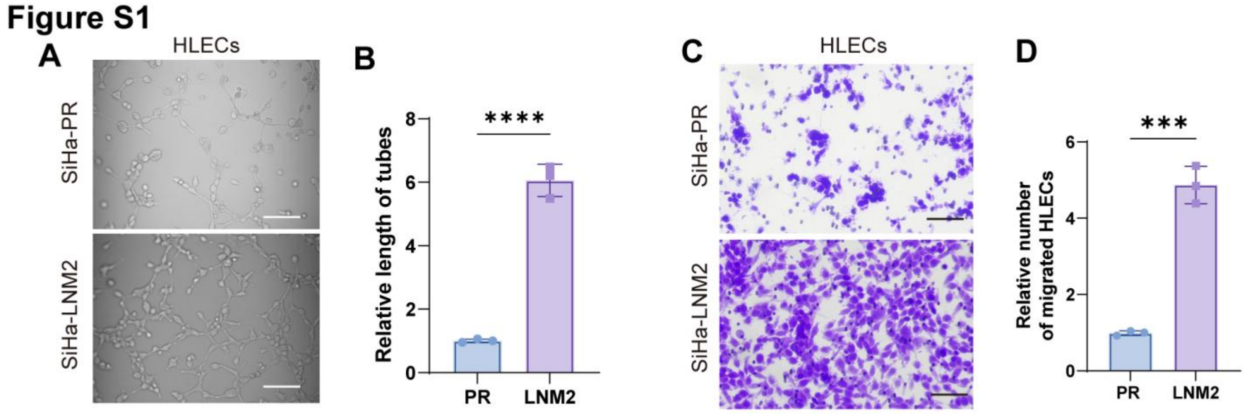


**Figure S1. Lymph node metastatic CSCC cells promotes HLECs tube formation and migration** A-B. Tube formation of HLECs treated with conditioned medium from SiHa-PR and SiHa-LNM2 cells. C-D. Transwell assays of HLECs treated with conditioned medium from SiHa-PR and SiHa-LNM2 cells. Each experiment was performed at least three times independently. ***P < 0.001; ****P < 0.0001.

Figure S2

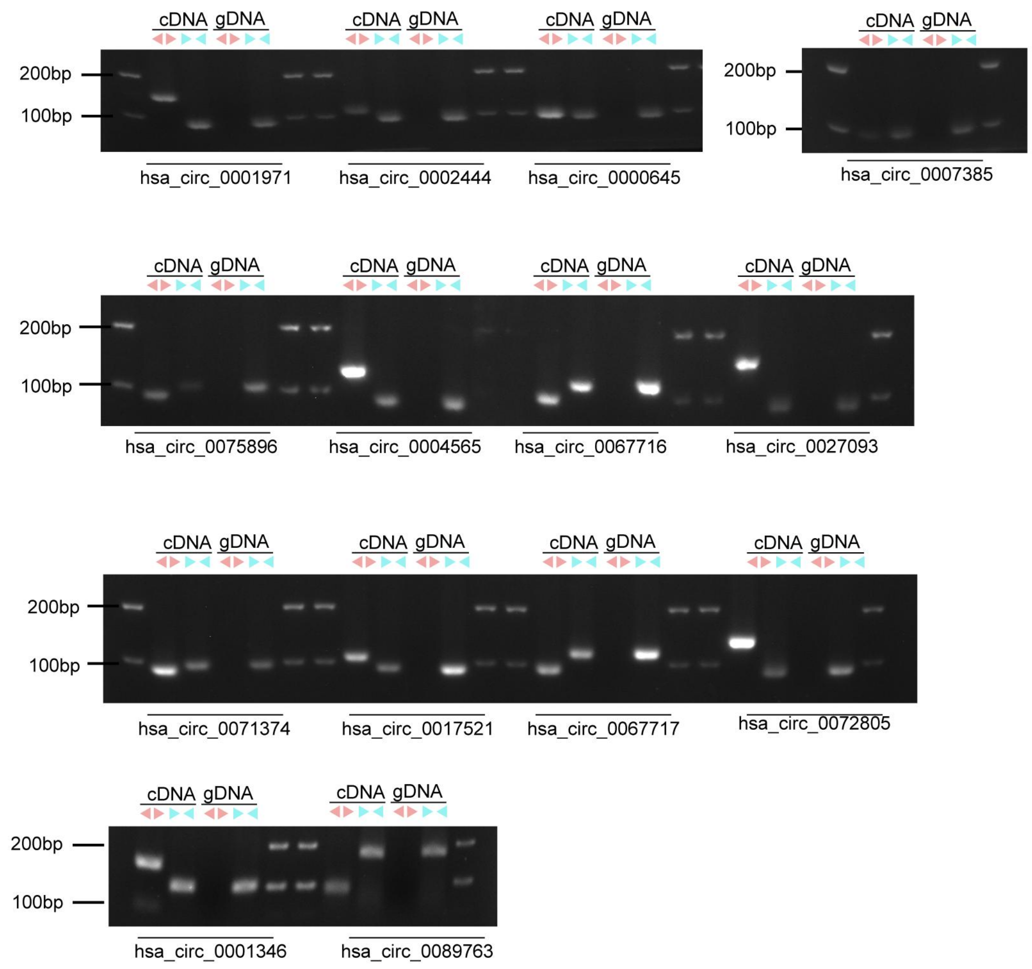


**Figure S2.** PCR with agarose gel electrophoresis assay indicated the presence of circular RNAs using convergent and divergent primers from cDNA or genomic DNA in CSCC cells.

**Figure S3**

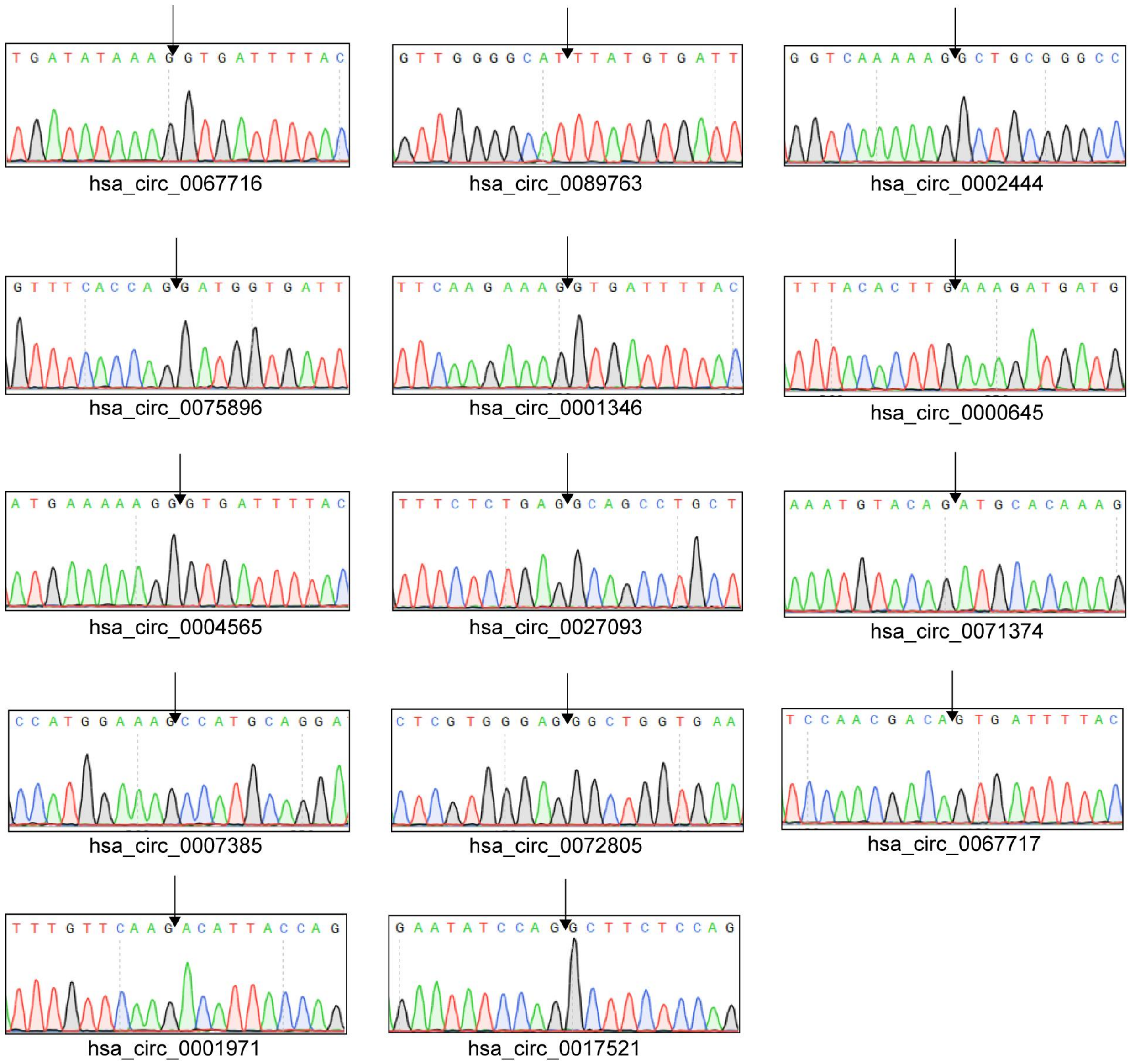


**Figure S3.** The back splicing junction of each circRNA was identified by Sanger sequencing.

Figure S4

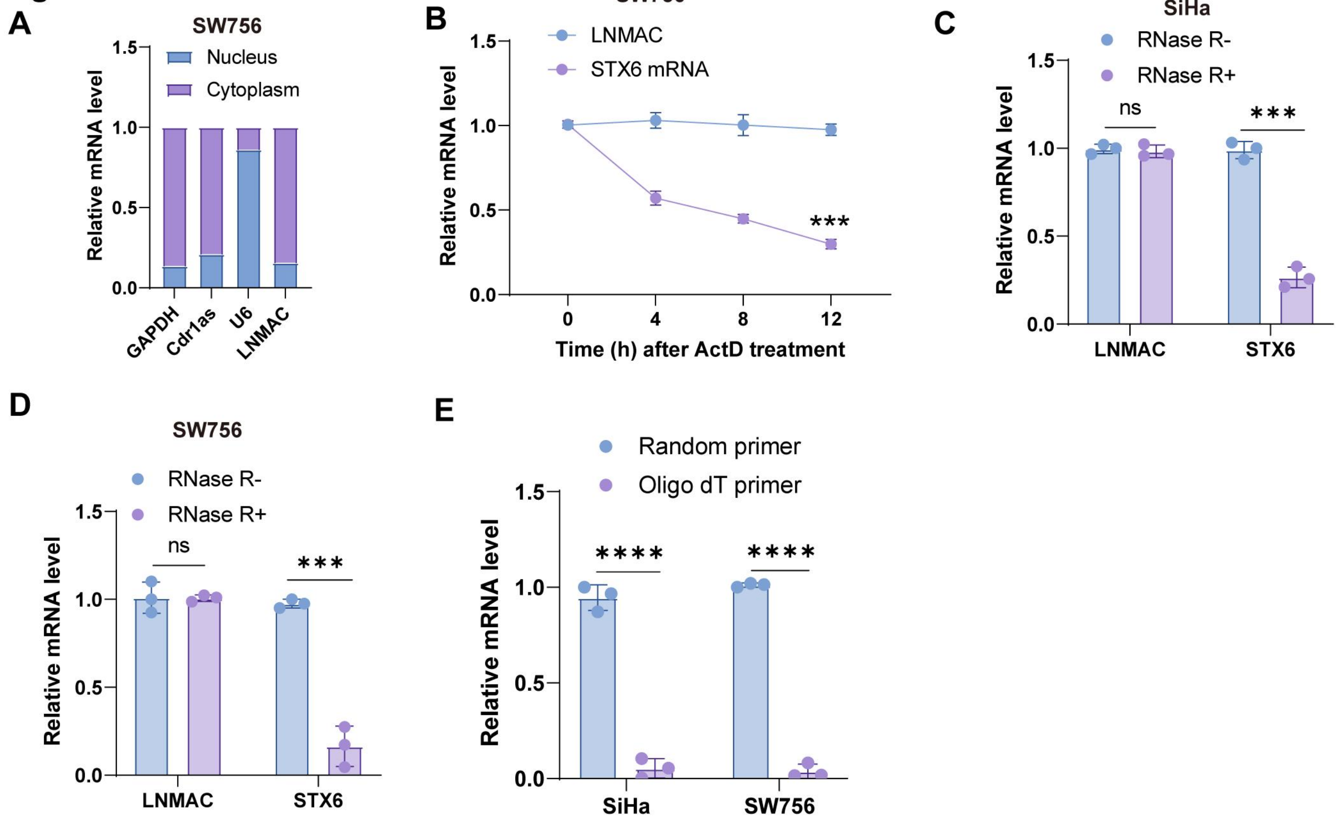


**Figure S4. Characterization of LNMAC in CSCC cells** A. The location of LNMAC was confirmed using a subcellular fractionation assay in SW756 cells. GAPDH and Cdr1as were used as controls which were mainly located in cytoplasm, while U6 was used as control which was mainly located nucleus. B. Actinomycin D assay was used to evaluate the stability of LNMAC and STX6 mRNA in SW756 cells. C-D. RT-qPCR analysis for the resistance of LNMAC and linear STX6 to RNase R in SiHa and SW756 cells. E. RT-qPCR analysis of LNMAC expression using random primers or oligo-dT primers in SiHa and SW756 cells. Each experiment was performed at least three times independently. ns no significance; ***P < 0.001; ****P < 0.0001.

Figure S5

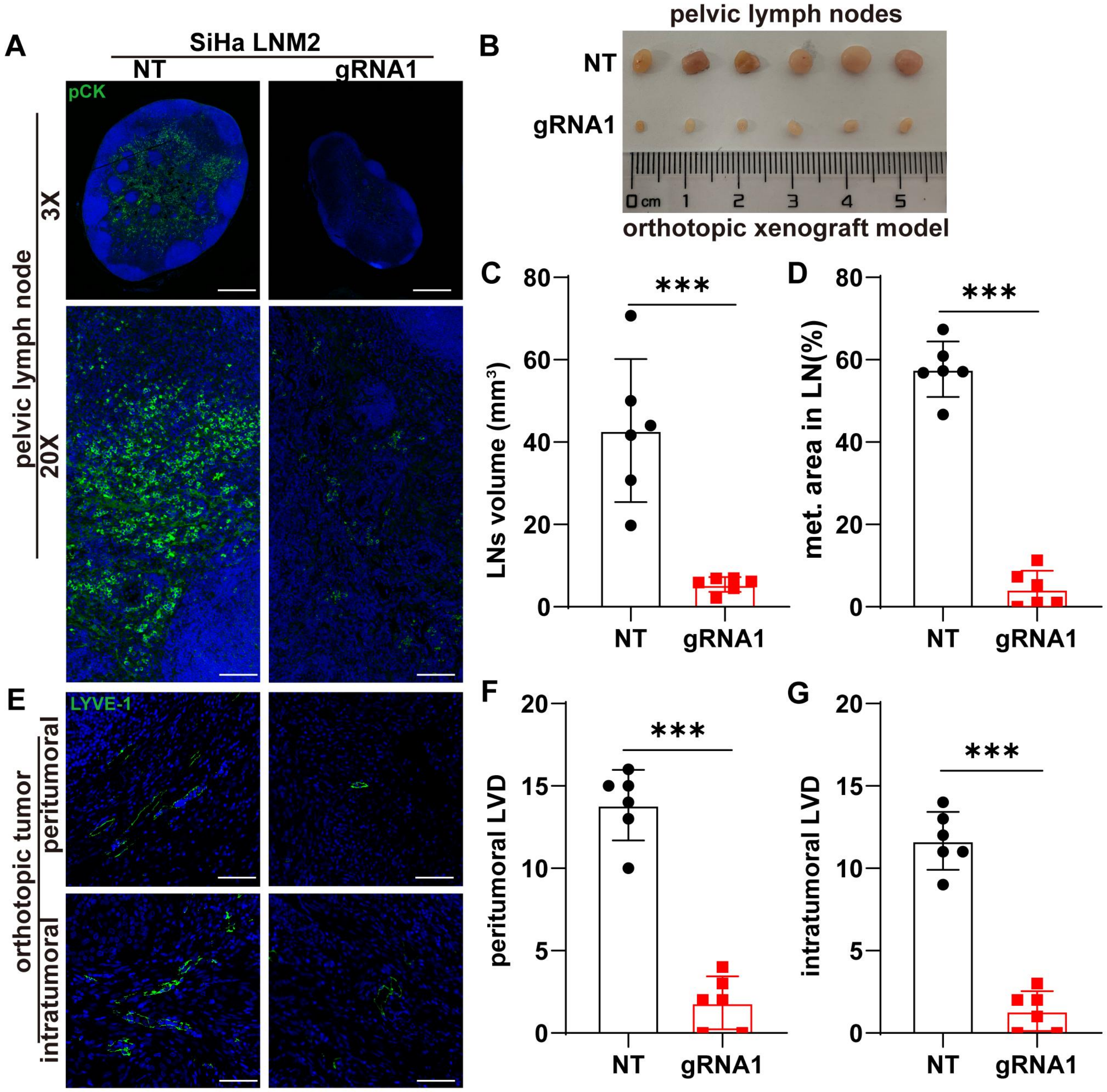


**Figure S5. LNMAC Promotes CSCC Lymph Node Metastasis in orthotopic xenograft model**

A-B. Immunofluorescent staining for pan-cytokeratin of pelvic LNs in different groups and the representative images of pelvic LNs in different groups (n=6). C. Pelvic LNs volumes in different groups. D. The metastatic area of pelvic LNs in each group. E-G. Quantification of lymphatic vascular density in peritumoral and intratumoral regions. ***P < 0.001.

Figure S6

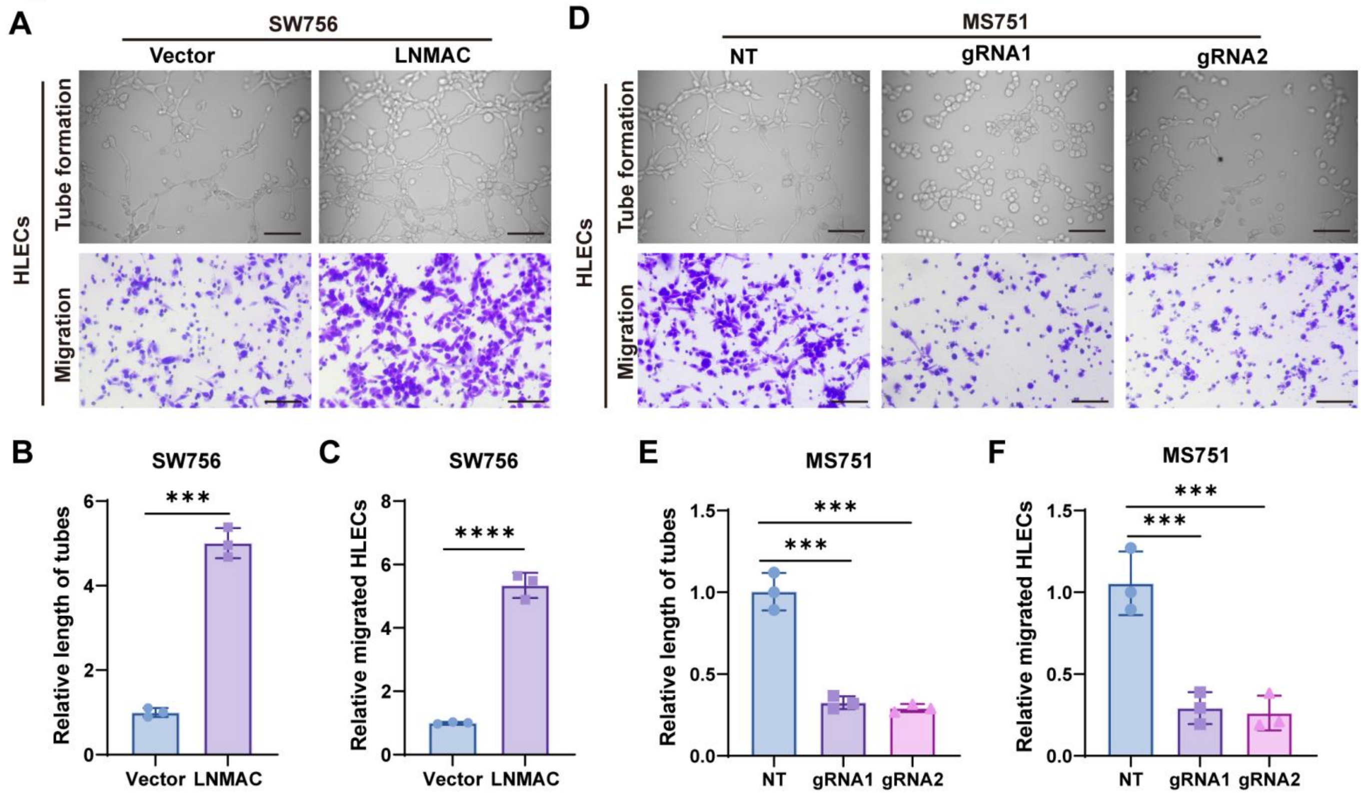


**Figure S6. LNMAC promotes lymphangiogenesis** A-C. Tube formation and transwell assays of HLECs treated with conditioned medium from LNMAC-overexpressing CSCC cells. D-F. Tube formation and transwell assays of HLECs treated with conditioned medium from LNMAC-silencing CSCC cells. Each experiment was performed at least three times independently. ***P < 0.001; ****P < 0.0001.

## Figure S7

**Peptides of HADC1 identified from Mass Spectrometry**

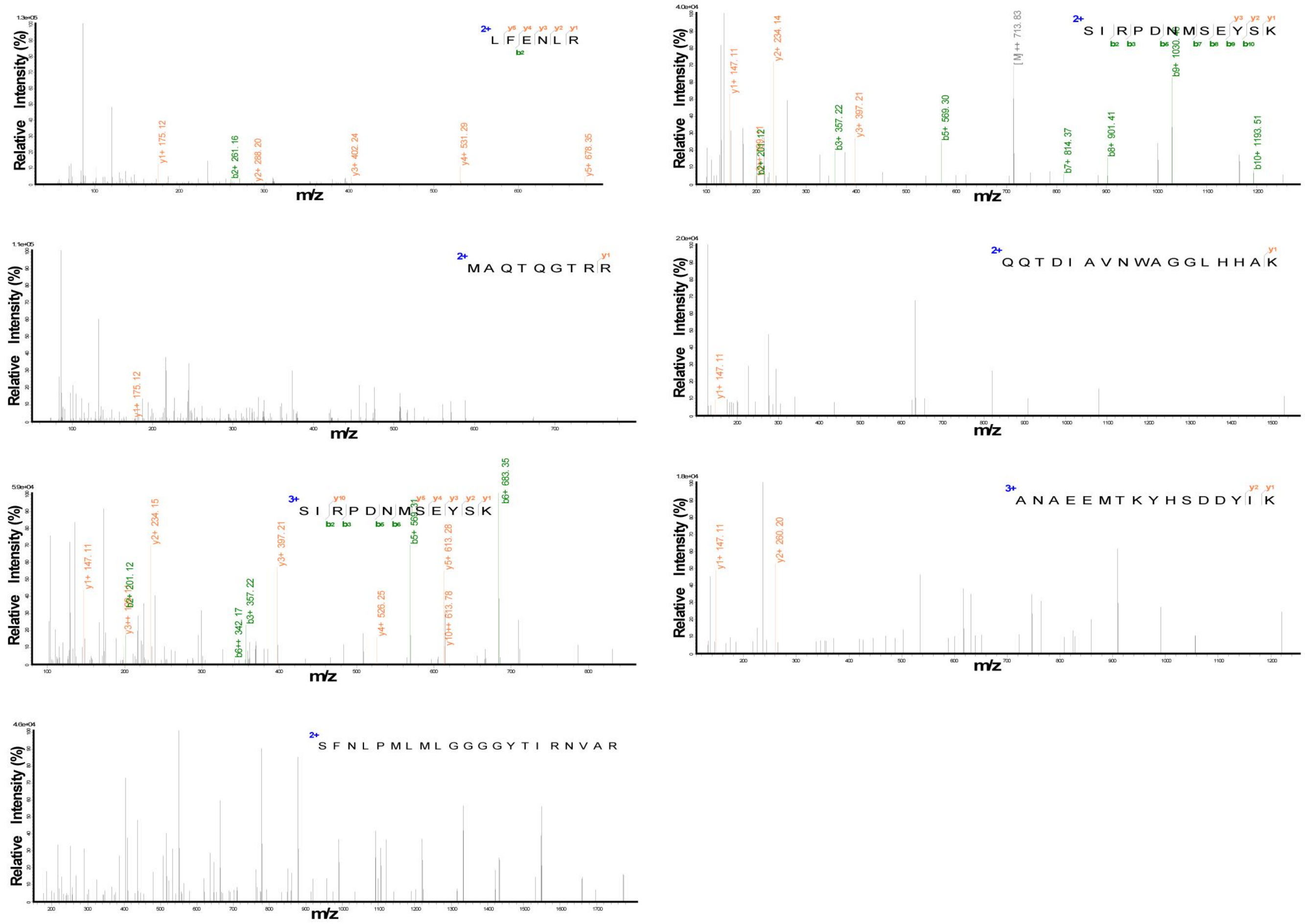


**Figure S7.** Mass spectrometry analysis of HDAC1 peptides after RNA pull-down assay.

## Figure S8

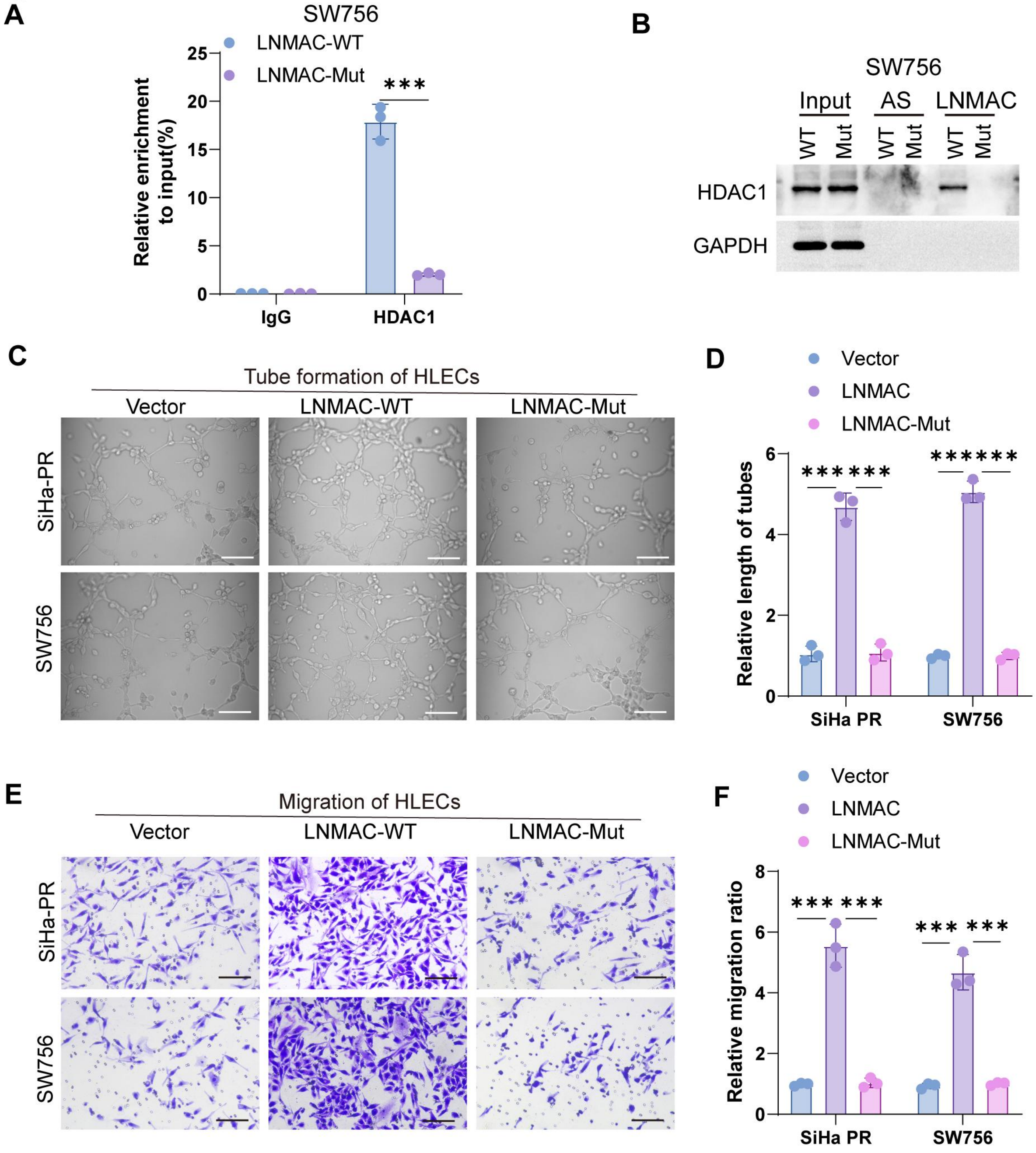


**Figure S8. LNMAC-induced lymphangiogenesis was dependent on direct interaction of LNMAC with HDAC1** A-B. RIP(A) and pull-down assay(B) showed the interaction between HDAC1 and LNMAC-WT, rather than LNMAC-Mut in SW756 cells. C-D. Tube formation of HLECs treated with conditioned medium from the indicated CSCC cells. E-F. Transwell assays of HLECs treated with conditioned medium from the indicated CSCC cells. Each experiment was performed at least three times independently. ns no significance; **P < 0.01; ***P < 0.001.

**Figure S9**

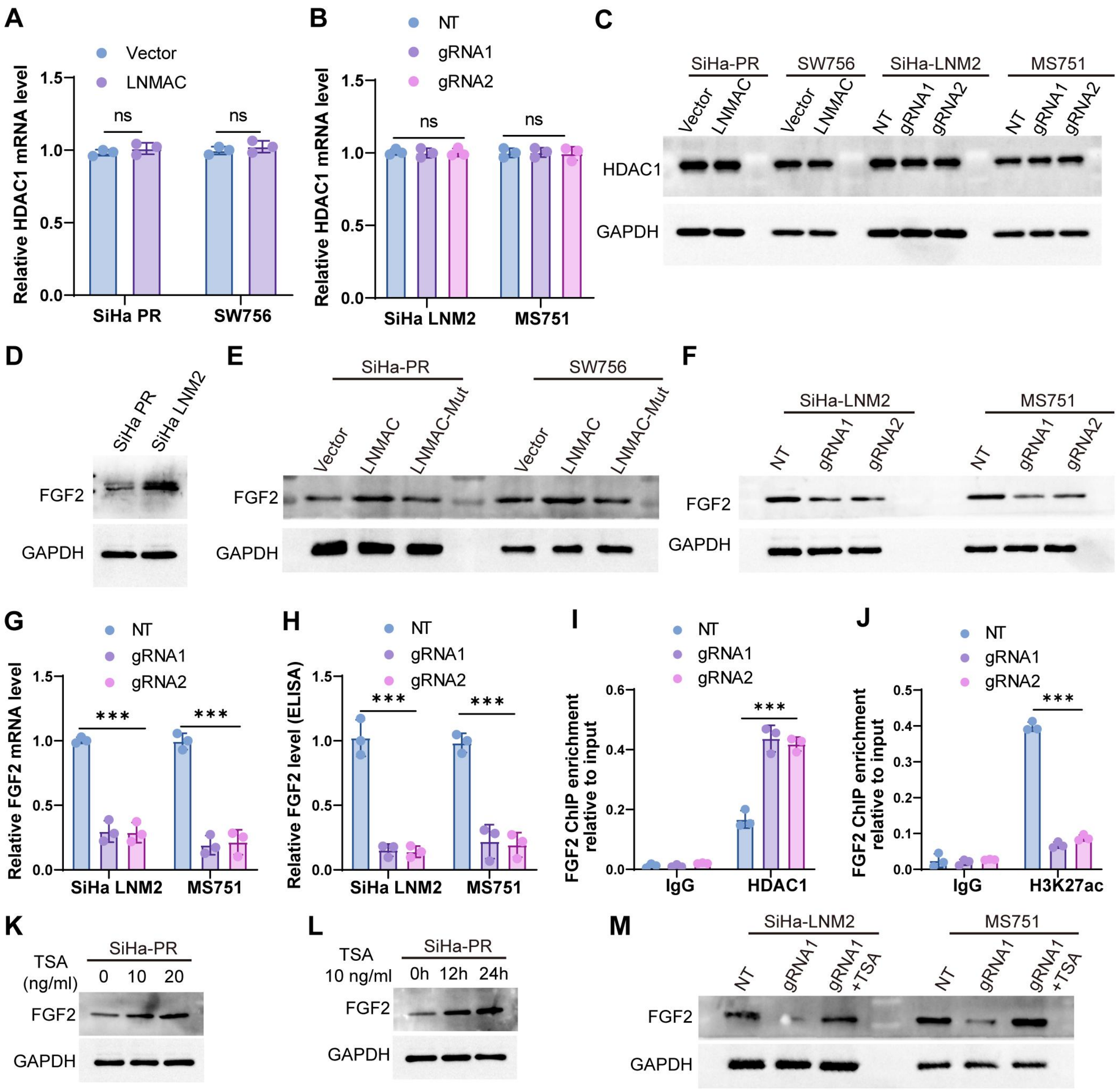


**Figure S9. HDAC1 is responsible for LNMAC mediated FGF2 expression** A-B. RT-qPCR analysis of HDAC1 mRNA expression after overexpressing or knocking down LNMAC. C. Western blotting analysis of HDAC1 protein expression after overexpressing or knocking down LNMAC in the indicated groups. D. FGF2 expression in poorly (SiHa-PR) and highly (SiHa-LNM2) metastatic CSCC cells. E-F. Western blotting analysis of FGF2 protein expression after overexpressing or knocking down LNMAC in the indicated groups. G-H. RT-qPCR and ELISA assay of FGF2 expression in LNMAC knockdown groups. I-J. Quantification of immunoprecipitated protein expression normalized by respective input in LNMAC knockdown groups. K-L. Western blotting analysis of FGF2 protein expression after treated with TSA at the indicted concentration and time point. M. Treating CSCC cells with TSA could recover FGF2 expression after LNMAC knockdown. Each experiment was performed at least three times independently. ns, no significance; ***P < 0.001.

**Figure S10**

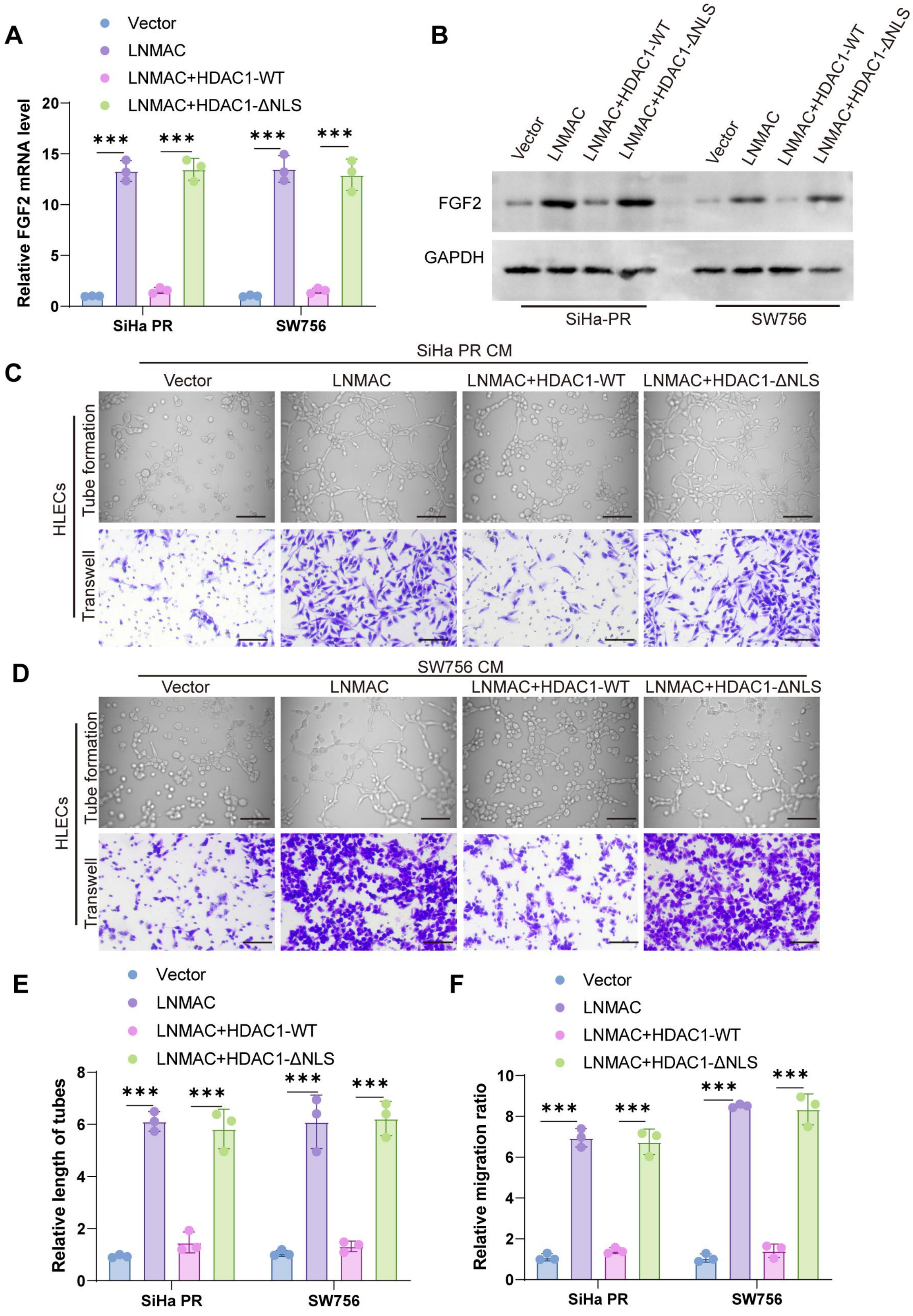


**Figure S10. Inhibiting HDAC1 nuclear translocation blocks LNMAC-induced lymphangiogenesis** A-B. RT-qPCR and western blotting analysis of FGF2 mRNA and protein expression in different groups. C-D. Representative images of tube formation and transwell assays of HLECs treated with conditioned medium from SiHa and SW756 cells. E-F. Statistical analysis of relative length of tubes and migration ratio in each group. Each experiment was performed at least three times independently. ***P < 0.001.

## Figure S11

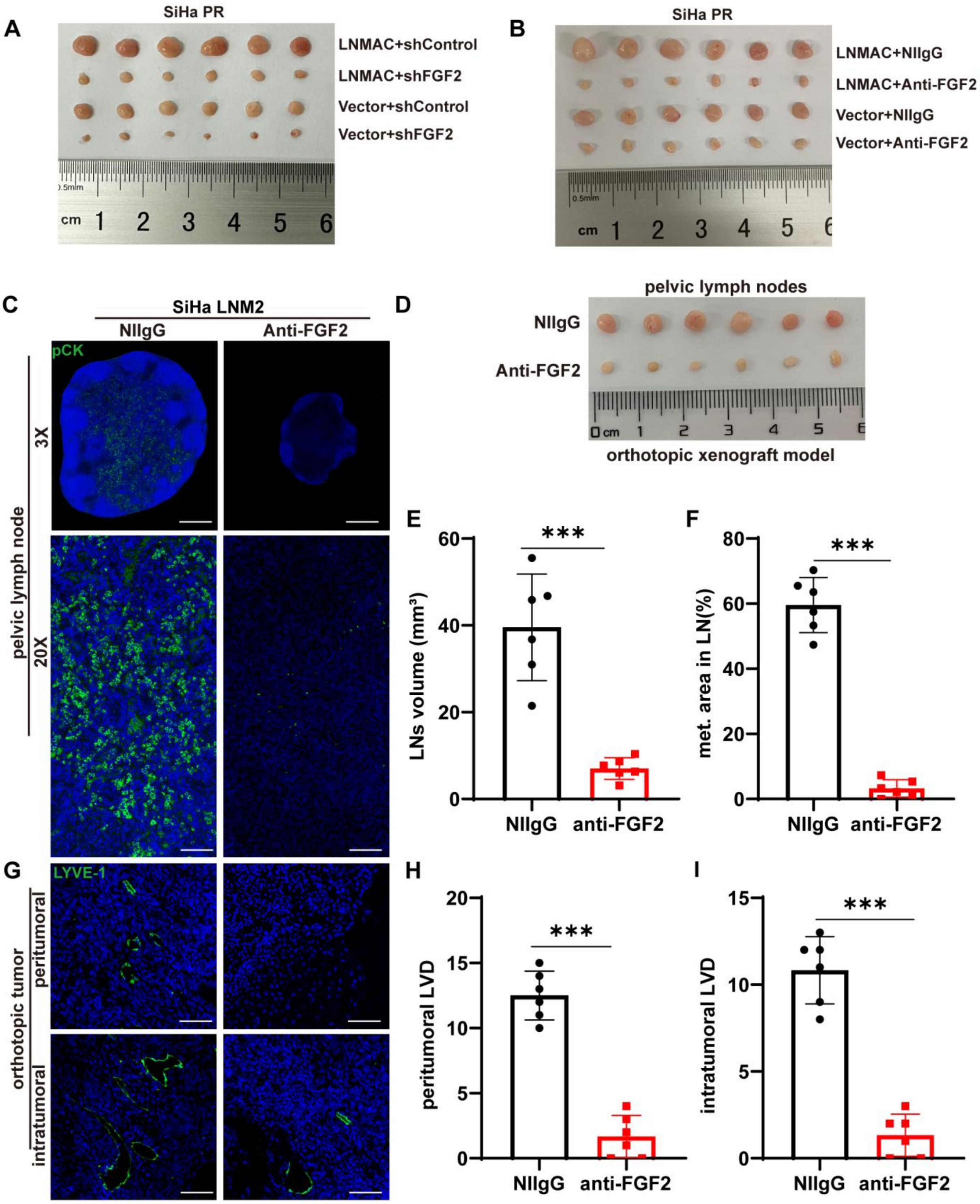


**Figure S11. Blocking FGF2 reverses LNMAC-induced Nodal Metastasis in vivo** A-B. Representative images of popliteal LNs (n=6) in the indicated groups in the footpad lymph node metastatic model. C-D. Immunofluorescent staining for pan-cytokeratin of pelvic LNs in different groups and the representative images of pelvic LNs in different groups (n=6) performed by orthotopic xenograft model. E. Pelvic LNs volumes in different groups. F. The metastatic area of pelvic LNs in each group. G-I. Quantification of lymphatic vascular density in peritumoral and intratumoral regions. ***P < 0.001.

**Supplementary Table S1. Antibodies used in this study**

| | | |
|---|---|---|
| FGF2 | Proteintech,#11234-1-AP | WB (1:1000) |
| LYVE-1 | Abcam, #ab219556 | IF and IHC (1:2000) |
| D2-40 | MAB-0567 | IHC (1:400) |
| Pan-Cytokeratin | CST, #4545S | IHC (1:400), IF (1:500) |
| GAPDH | Proteintech,#10494-1-AP | WB (1:10000) |
| H3K27ac | ZENBIO, #R26244 | ChIP (1:20) |
| HDAC1 | Proteintech,#10197-1-AP | WB (1:10000),IF/IP (1:600) |
| Donkey anti-Rabbit IgG (H+L) Highly Cross-Adsorbed Secondary Antibody, Alexa Fluor™ 488 | ThermoFisher, #A21206 | IF (1:400) |
| HRP-conjugated Affinipure Goat Anti-Rabbit IgG(H+L) | Proteintech, #SA00001-2 | WB (1:10000) |
| HRP-conjugated Affinipure Goat Anti-Mouse IgG(H+L) | Proteintech, #SA00001-1 | WB (1:10000) |
| Flag | Sigma-Aldrich, #F3165 | WB(1:5000) |

**Supplementary Table S2. Sequences of primers, gRNAs and probes**

| | |
|---|---|
| **Primers for RT-qPCR and RIP-qPCR** | |
| FGF2 | F 5'-AGAAGAGCGACCCTCACATCA-3' |
| | R 5'-CGGTTAGCACACACTCCTTTG-3' |
| C-GAPDH | F 5'-AGAAGGCTGGGGCTCATTTG-3' |
| | R 5'-AGGGGCCATCCACAGTCTTC-3' |
| CDR1as | F 5'-ACGTCTCCAGTGTGCTGA-3' |
| | R 5'-CTTGACACAGGTGCCATC-3' |
| D-GAPDH | F 5'-TGTACCATCAATAAAGTACCCTGTG-3' |
| | R 5'-AAATCCGTTGACTCCGACCT-3' |
| U6 | F 5'-ACAGATCTGTCGGTGTGGCAC-3' |
| | R 5'-GGCCCCGGATTATCCGACATTC-3' |
| D-hsa_circ_0001971 | F 5'-ACAATGCTGCCTTAACTTAC-3' |
| | R 5'-TTGTGGCTCCTGGATAAC-3' |
| C-hsa_circ_0001971 | F 5'-GTTATCCAGGAGCCACAA-3' |
| | R 5'-CTCTCCACTGCGATAGAAT-3' |
| D-hsa_circ_0002444 | F 5'-TTCTGAGACAACGATGGT-3' |
| | R 5'-TCCTGCCAAGATGAGATT-3' |
| C-hsa_circ_0002444 | F 5'-CTGAAATTGCCAAGAATCTC-3' |
| | R 5'-CTTCTGGAGTTACCTGTTC-3' |
| D-hsa_circ_0000645 | F 5'-ATGGAAATGTGACTGTGTTG-3' |
| | R 5'-GTCTCTTGTGCTCTGGAA-3' |
| C-hsa_circ_0000645 | F 5'-TGATGACATTGAAGAAGGAG-3' |
| | R 5'-GCTTGGGTCTATCTTTGAG-3' |
| D-hsa_circ_0007385 | F 5'-GCATTTGCCTTATACAGC-3' |
| | R 5'-CACTTGTTTATAAGCATGGG-3' |
| C-hsa_circ_0007385 | F 5'-GACCTTCGTATTGACCAA-3' |
| | R 5'-CTGTGTTCATCTTCATCTG-3' |
| D-hsa_circ_0075896 | F 5'-TGTAAGACATCACAATCCCA-3' |
| | R 5'-TGAGCAATCACCATCCTG-3' |
| C-hsa_circ_0075896 | F 5'-TCTTGGTATCGCAACTAC-3' |
| | R 5'-CTCTGAATAATGGGATTGTG-3' |
| D-hsa_circ_0004565 | F 5'-CAACGACATTGAGGTACTAA-3' |
| | R 5'-CTATGGAGAGCAGCATCT-3' |
| C-hsa_circ_0004565 | F 5'-TTACAACGAGATGCTGCT-3' |
| | R 5'-CTGGACAGTCAAGATGGT-3' |
| D-hsa_circ_0067716 | F 5'-GACAATTCATCTGGCACTT-3' |
| | R 5'-CTATGGAGAGCAGCATCT-3' |
| C-hsa_circ_0067716 | F 5'-CACAAGTCTACACCATCT-3' |
| | R 5'-GGAGGTCATCAAATGTCT-3' |
| D-hsa_circ_0027093 | F 5'-GCTTAATTGGCTTAGTGTC-3' |
| | R 5'-CATCCTTACTGTCTTCAAC-3' |
| C-hsa_circ_0027093 | F 5'-TTGTGTTGAAGACAGTAAGG-3' |
| | R 5'-TTATCACTTCCTCCGAGAC-3' |
| D-hsa_circ_0071374 | F 5'-TGCAGAAATTCAGGTTACAG-3' |
| | R 5'-CTTTTGAATCGAGAGGCTTCT-3' |
| C-hsa_circ_0071374 | F 5'-TTACAGGTGAGATGAGTGA-3' |
| | R 5'-AATCAATTAGCATCCTCAGG-3' |
| D-hsa_circ_0017521 | F 5'-GAATATCCAGGCTTCTCC-3' |
| | R 5'-CCCTCTCATTGTGGTAAA-3' |
| C-hsa_circ_0017521 | F 5'-CAATCCAGAGATGTGTCC-3' |
| | R 5'-GGTTGTCTATCAGGTCAA-3' |
| D-hsa_circ_0067717 | F 5'-AAGGCAGCCATAGTTCAC-3' |

| | |
|---|---|
| | R 5'-AGCAGCATCTCGTTGTAA-3' |
| C-hsa_circ_0067717 | F 5'-CACAAGTCTACACCATCT-3' |
| | R 5'-GGAGGTCATCAAATGTCT-3' |
| D-hsa_circ_0072805 | F 5'-TACCTGATGCACGAACAC-3' |
| | R 5'-TCCACGACTTTGTTTTCTG-3' |
| C-hsa_circ_0072805 | F 5'-GAATTACCAGATCTCCGTCAAG-3' |
| | R 5'-CCTGGTTGTCCACGACTT-3' |
| D-hsa_circ_0001346 | F 5'-GTCCAGGATAGACATAGAG-3' |
| | R 5'-ACAGTCAAGATGGTGTAG-3' |
| C-hsa_circ_0001346 | F 5'-ACCATCTTGACTGTCCAG-3' |
| | R 5'-AGGGAGGTCATCAAATGTC-3' |
| D-hsa_circ_0089763 | F 5'-GGTGATGAGGAATAGTGTAAG-3' |
| | R 5'-ATCCTAACCCTACTCCTAATC-3' |
| C-hsa_circ_0089763 | F 5'-TATGAGGTGTGAGCGATA-3' |
| | R 5'-GCCTATGAGTGACTACAA-3' |
| D-LNMAC | F 5'-TGGAGGAACAGGCAGTTATGTTG-3' |
| | R 5'-TTGACATCTGATCTTTCATGTCCAC-3' |
| C-LNMAC | F 5'-CACTGGAACAACAGATAAATATGGG-3' |
| | R 5'-TGCTGCTCCTCAATGAAATGA-3' |
| STX6 | F 5'-CACCAACGAGCTGAGAAATAACC-3' |
| | R 5'-CCCTGACAACTTGCCGAGT-3' |
| **Primers for ChIP-qPCR** | |
| FGF2 | F 5'-AGACTCTTCCTTGGATTGC-3' |
| | R 5'-CAGCCGTGACTCAACTTT-3' |
| **sgRNA sequences** | |
| NT-F | AAACGCAGGGTTTTCCCAGTCACGACGTTGTAAA |
| NT-R | AAAATTTACAACGTCGTGACTGGGAAAACCCTGC |
| gRNA1-F | CTGATCTTTCATGTCCACTGGTCATATGAG |
| gRNA1-R | CTCATATGACCAGTGGACATGAAAGATCAG |
| gRNA2-F | GACATCTGATCTTTCATGTCCACTGGTCAT |
| gRNA2-R | ATGACCAGTGGACATGAAAGATCAGATGTC |
| **shRNA sequences** | |
| shFGF2 | ACTACAATACTTACCGGTCAA |
| **siRNA sequences** | |
| si-Control | UUCUCCGAACGUGUCACGU |
| si-Importin-α1 | UCAUGUAGCUGAGACAUAA |
| si-Importin-α3 | GCCCUCUCUUACCUUACUG |
| si-Importin-α4 | UUGUCCUCCACAAACAUAU |
| si-Importin-α5 | GCCUUUGAUCUUAUUGAGC |
| si-Importin-α6 | CAGUUGUUCAAACGCAGAA |
| si-Importin-α7 | CGGAGAAAUGUGGAGCUGA |
| si-Importin-α8 | GCUGCAUGAGAACCGUCAA |
| **ISH probes** | |
| Digoxigenin-labeled LNMAC probe for ISH | 5'-CTGATCTTTCATGTCCACTGGTCATATGAGATA-3' |
| Cy3-labeled LNMAC probe for FISH | 5'-ATCTTTCATGTCCACTGGTCAT-3' |
| **Primers for *in vitro* transcription** | |
| LNMAC(1-391)-F | 5'-TAATACGACTCACTATAGGGGACATGAAAGATCAGATGTC-3' |
| LNMAC(1-391)-R | 5'-CACTGGTCATATGAGATAC-3' |
| LNMAC(1-150)-F | 5'-TAATACGACTCACTATAGGGGACATGAAAGATCAGATGTC-3' |
| LNMAC(1-150)-R | 5'-GGCTCTCTGGAGCTCTCGGTC-3' |
| LNMAC(1-90)-F | 5'-TAATACGACTCACTATAGGGGACATGAAAGATCAGATGTC-3' |

| | |
|---|---|
| LNMAC(1-90)-R | 5'-CTGGCTGCCACTGTCTCCCAG-3' |
| LNMAC(90-150)-F | 5'-TAATACGACTCACTATAGGGGAACTGGAGCACTGGAACAAC-3' |
| LNMAC(90-150)-R | 5'-GGCTCTCTGGAGCTCTCGGTC-3' |
| LNMAC(150-391)-F | 5'-TAATACGACTCACTATAGGGCAATTCTCATTTCATTGAGG-3' |
| LNMAC(150-391)-R | 5'-CACTGGTCATATGAGATAC-3' |
| LNMAC(150-270)-F | 5'-TAATACGACTCACTATAGGGCAATTCTCATTTCATTGAGG-3' |
| LNMAC(150-270)-R | 5'-GATGCGCTGGGACATGTTC-3' |
| LNMAC(270-391)-F | 5'-TAATACGACTCACTATAGGGCGGAGGGGAGCTGGAGGAAC-3' |
| LNMAC(270-391)-R | 5'-CACTGGTCATATGAGATAC-3' |
| LNMAC(Antisense)-F | 5'-TAATACGACTCACTATAGGGGTGACCAGTATACTCTATG-3' |
| LNMAC(Antisense)-R | 5'-CTGTACTTTCTAGTCTACAG-3' |

**Supplementary Table S3. LNMAC-interacting proteins identified by MS**

| Protein Name | score | mass | | emPAI |
|---|---|---|---|---|
| sp\|Q13547\|HDAC1_HUMAN | 259 | 55638 | | 1.22 |
| sp\|P68400\|CSK21_HUMAN | 39 | 45229 | | 1.02 |
| sp\|P51858\|HDGF_HUMAN | 40 | 26886 | | 0.26 |
| sp\|Q5JXB2\|UE2NL_HUMAN | 59 | 17366 | | 0.2 |
| sp\|P32320\|CDD_HUMAN | 27 | 16687 | | 0.2 |
| sp\|Q3SY84\|K2C71_HUMAN | 44 | 57769 | | 0.18 |
| sp\|P83916\|CBX1_HUMAN | 51 | 21519 | | 0.16 |
| sp\|Q5BJF2\|SGMR2_HUMAN | 34 | 21005 | | 0.16 |
| sp\|P00568\|KAD1_HUMAN | 48 | 21735 | | 0.15 |
| sp\|Q9H0U4\|RAB1B_HUMAN | 29 | 22328 | | 0.15 |
| sp\|Q9BVA1\|TBB2B_HUMAN | 80 | 50377 | | 0.14 |
| sp\|Q8TEA8\|DTD1_HUMAN | 65 | 23580 | | 0.14 |
| sp\|P33316\|DUT_HUMAN | 44 | 26832 | | 0.12 |
| sp\|P31944\|CASPE_HUMAN | 44 | 27947 | | 0.12 |
| sp\|Q14847\|LASP1_HUMAN | 36 | 30097 | | 0.11 |
| sp\|Q53GQ0\|DHB12_HUMAN | 37 | 34416 | | 0.1 |
| sp\|P06493\|CDK1_HUMAN | 25 | 34131 | | 0.1 |
| sp\|O95433\|AHSA1_HUMAN | 40 | 38421 | | 0.09 |
| sp\|Q6PK18\|OGFD3_HUMAN | 37 | 35852 | | 0.09 |
| sp\|P12429\|ANXA3_HUMAN | 24 | 36524 | | 0.09 |
| sp\|O15527\|OGG1_HUMAN | 24 | 39214 | | 0.08 |
| sp\|Q6YI46\|TMM64_HUMAN | 14 | 40266 | | 0.08 |
| sp\|P05455\|LA_HUMAN | 41 | 46979 | | 0.07 |
| sp\|Q9UKU7\|ACAD8_HUMAN | 36 | 45896 | | 0.07 |
| sp\|Q14240\|IF4A2_HUMAN | 36 | 46601 | | 0.07 |
| sp\|Q9UQ80\|PA2G4_HUMAN | 27 | 44101 | | 0.07 |
| sp\|P17706\|PTN2_HUMAN | 26 | 48842 | | 0.07 |
| sp\|Q6BAA4\|FCRLB_HUMAN | 23 | 47274 | | 0.07 |
| sp\|Q16204\|CCDC6_HUMAN | 46 | 53429 | | 0.06 |
| sp\|Q9HD26\|GOPC_HUMAN | 34 | 50888 | | 0.06 |
| sp\|P40227\|TCPZ_HUMAN | 32 | 58444 | | 0.06 |
| sp\|P36871\|PGM1_HUMAN | 30 | 61696 | | 0.05 |
| sp\|Q10471\|GALT2_HUMAN | 28 | 65433 | | 0.05 |
| sp\|Q15642\|CIP4_HUMAN | 28 | 68538 | | 0.05 |
| sp\|P30291\|WEE1_HUMAN | 27 | 72237 | | 0.05 |
| sp\|Q9NRF8\|PYRG2_HUMAN | 22 | 66320 | | 0.05 |
| sp\|P42166\|LAP2A_HUMAN | 53 | 76016 | | 0.04 |
| sp\|Q9NQI0\|DDX4_HUMAN | 39 | 80113 | | 0.04 |
| sp\|P21980\|TGM2_HUMAN | 31 | 78420 | | 0.04 |
| sp\|P02545\|LMNA_HUMAN | 24 | 74380 | | 0.04 |
| sp\|Q08945\|SSRP1_HUMAN | 18 | 81367 | | 0.04 |
| sp\|O00203\|AP3B1_HUMAN | 55 | 121815 | | 0.03 |
| sp\|P16615\|AT2A2_HUMAN | 26 | 116336 | | 0.03 |
| sp\|Q02413\|DSG1_HUMAN | 25 | 114702 | | 0.03 |
| sp\|Q06418\|TYRO3_HUMAN | 24 | 97927 | | 0.03 |
| sp\|Q6AI08\|HEAT6_HUMAN | 22 | 130410 | | 0.03 |
| sp\|P0C874\|S31D3_HUMAN | 21 | 103380 | | 0.03 |
| sp\|P15924\|DESP_HUMAN | 72 | 334021 | | 0.02 |
| sp\|O14617\|AP3D1_HUMAN | 59 | 131159 | | 0.02 |
| sp\|O94759\|TRPM2_HUMAN | 28 | 172743 | | 0.02 |
| sp\|Q9H9B1\|EHMT1_HUMAN | 27 | 144000 | | 0.02 |

| | | | | |
|---|---|---|---|---|
| sp\|Q9C0B2\|CFA74_HUMAN | 25 | 179846 | | 0.02 |
| sp\|Q9HCE6\|ARGAL_HUMAN | 24 | 141887 | | 0.02 |
| sp\|Q7Z406\|MYH14_HUMAN | 31 | 228701 | | 0.01 |
| sp\|Q9P225\|DYH2_HUMAN | 31 | 510796 | | 0.01 |
| sp\|Q5TH69\|BIG3_HUMAN | 22 | 243862 | | 0.01 |

**Supplementary Table S4. Correlation between LNMAC expression and clinicopathologic characteristics of CSCC**

| Characteristics | Total | LNMAC expression | | P value |
|---|---|---|---|---|
| | 201 | High | Low | |
| Age (years) | | | | 0.7791 |
| < 42 | 52 | 27 | 25 | |
| ≥ 42 | 149 | 74 | 75 | |
| FIGO stage | | | | 0.8043 |
| Ⅰ (Ⅰa2 + Ⅰb1 + Ⅰb2) | 133 | 66 | 67 | |
| Ⅱ (Ⅱa1 + Ⅱa2) | 68 | 35 | 33 | |
| Tumor size (cm) | | | | 0.4712 |
| ≤ 4 | 140 | 68 | 72 | |
| > 4 | 61 | 33 | 28 | |
| Differentiation | | | | 0.6312 |
| Well | 39 | 19 | 20 | |
| Moderate | 59 | 27 | 32 | |
| Poor | 103 | 55 | 48 | |
| Stromal invasion | | | | 0.2595 |
| < 1/2 | 154 | 74 | 80 | |
| ≥ 1/2 | 47 | 27 | 20 | |
| LVSI | | | | <0.0001 |
| Positive | 58 | 48 | 10 | |
| Negative | 143 | 53 | 90 | |
| LNM | | | | <0.0001 |
| Positive | 52 | 41 | 11 | |
| Negative | 149 | 60 | 89 | |
| Vaginal invasion | | | | 0.6370 |
| Positive | 18 | 10 | 8 | |
| Negative | 183 | 91 | 92 | |
| Parametrial invasion | | | | 0.9846 |
| Positive | 14 | 7 | 7 | |
| Negative | 187 | 94 | 93 | |

$\chi^2$-test. FIGO: the International Federation of Gynecology and Obstetrics; LVSI, lymphovascular space invasion; LNM, lymph node metastasis.
